# NAD^+^ biosynthesis in bacteria is controlled by global carbon/nitrogen levels via PII signaling

**DOI:** 10.1074/jbc.RA120.012793

**Published:** 2020-03-16

**Authors:** Adrian Richard Schenberger Santos, Edileusa Cristina Marques Gerhardt, Erick Parize, Fabio Oliveira Pedrosa, Maria Berenice Reynaud Steffens, Leda Satie Chubatsu, Emanuel Maltempi Souza, Luciane Maria Pereira Passaglia, Fernando Hayashi Sant'Anna, Gustavo Antônio de Souza, Luciano Fernandes Huergo, Karl Forchhammer

**Affiliations:** ‡ Departamento de Bioquímica e Biologia Molecular, Universidade Federal do Paraná (UFPR), Curitiba, Paraná, CEP: 81531-980 Brazil; ** Setor Litoral, UFPR, Matinhos, Paraná, CEP: 83260-000 Brazil; § Interfakultäres Institut für Mikrobiologie und Infektionsmedizin der Eberhard-Karls Universität Tübingen, Auf der Morgenstelle 28, Tübingen 72076, Germany; ¶ Departamento de Genética, Instituto de Biociências, Universidade Federal do Rio Grande do Sul, Porto Alegre, CEP:91501-970 CP 15053 Brazil; ‖ Departamento de Bioquímica, Universidade Federal do Rio Grande do Norte, Natal/RN, CEP: 59072-970 Brazil

**Keywords:** metabolic regulation, nicotinamide adenine dinucleotide (NAD), protein-protein interaction, allosteric regulation, bacterial metabolism, 2-oxoglutarate (2-OG), NadE, nutrient sensing, PII protein

## Abstract

NAD^+^ is a central metabolite participating in core metabolic redox reactions. The prokaryotic NAD synthetase enzyme NadE catalyzes the last step of NAD^+^ biosynthesis, converting nicotinic acid adenine dinucleotide (NaAD) to NAD^+^. Some members of the NadE family use l-glutamine as a nitrogen donor and are named NadE^Gln^. Previous gene neighborhood analysis has indicated that the bacterial *nadE* gene is frequently clustered with the gene encoding the regulatory signal transduction protein PII, suggesting a functional relationship between these proteins in response to the nutritional status and the carbon/nitrogen ratio of the bacterial cell. Here, using affinity chromatography, bioinformatics analyses, NAD synthetase activity, and biolayer interferometry assays, we show that PII and NadE^Gln^ physically interact *in vitro*, that this complex relieves NadE^Gln^ negative feedback inhibition by NAD^+^. This mechanism is conserved in distantly related bacteria. Of note, the PII protein allosteric effector and cellular nitrogen level indicator 2-oxoglutarate (2-OG) inhibited the formation of the PII-NadE^Gln^ complex within a physiological range. These results indicate an interplay between the levels of ATP, ADP, 2-OG, PII-sensed glutamine, and NAD^+^, representing a metabolic hub that may balance the levels of core nitrogen and carbon metabolites. Our findings support the notion that PII proteins act as a dissociable regulatory subunit of NadE^Gln^, thereby enabling the control of NAD^+^ biosynthesis according to the nutritional status of the bacterial cell.

## Introduction

**Figure 1.**
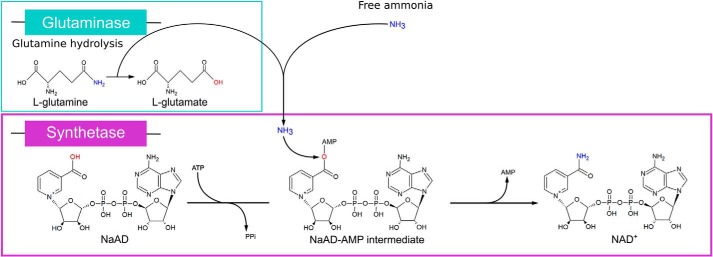
**Reaction scheme of NAD synthetases.** Last step of the NAD^+^ biosynthesis catalyzed by NadE using l-glutamine or free ammonia as N donor for the amidation of the precursor NaAD. The hydrolysis of l-glutamine occurs in the glutaminase domain (in *cyan*). The amidation of NaAD is performed by the synthetase domain (in *purple*).

NAD^+^ is a crucial metabolite participating as a cofactor in dozens of core metabolic redox reactions. Furthermore, NAD^+^ is used as substrate for lysine deacetylases, ADP-ribosylating enzymes, DNA ligase, and for NADP^+^ biosynthesis. Increasing evidence supports that NAD^+^ also acts as an important signaling metabolite and that NAD^+^ is used for 5′-modified mRNA forming a caplike structure in bacteria (1–3). In prokaryotes, NAD^+^ biosynthesis can occur by more than one route, including *de novo*, using l-aspartate as precursor, and salvage pathways. In most cases, these routes converge before the final reaction, catalyzed by NAD synthetase enzymes (NadE) in which nicotinic acid adenine dinucleotide (NaAD) is amidated to produce NAD^+^ (4). The NadE enzymes use ATP as a substrate to produce an NaAD-AMP intermediate, thereby activating the carboxylate of nicotinic acid to react with ammonia, releasing NAD^+^, AMP, and PP_i_ as products (Fig. 1).

There are two different classes of NadE enzymes identified to date. A shorter version of the enzymes carries only the NAD synthetase domain and uses ammonia directly as N donor. These enzymes are known as ammonia dependent or NadE^NH3^. Longer versions of NadE enzymes carry an extra N-terminal glutaminase domain and use glutamine as N donor to deliver ammonia to the synthetase domain through an ammonia tunnel (5, 6) (Fig. 1). These enzymes are known as glutamine dependent or NadE^Gln^. Regarding the quaternary structure arrangement, NadE^NH3^ enzymes are found as dimers, whereas NadE^Gln^ exists in two different subtypes to date: type 1 is octamers, and type 2 is dimers. Kinetic and structural analysis suggests that the dimeric type 2 NadE^Gln^ is an evolutionary intermediate between dimeric NadE^NH3^ and octameric, or type 1 NadE^Gln^ (7).

There is very little knowledge regarding the regulation of NAD^+^ biosynthesis pathways in bacteria. In *Salmonella enterica*, the expression of the enzymes involved in *de novo* NAD^+^ biosynthesis is regulated by NadR, which is an NAD^+^ sensor repressing the biosynthesis of NAD^+^ when the levels of this metabolite are high (8). There are other transcriptional regulators of NAD^+^ biosynthesis described in bacteria. However, their sensory properties and mode of action have not been studied in detail (9).

The conservation of gene order can be used as a fingerprint of proteins that physically interact (10). In prokaryotes, the gene encoding NadE^Gln^ is frequently co-localized with the gene encoding the regulatory signal transduction protein PII (usually termed *glnB*) (11). Conservation of the *glnB-nadE* gene pair leads to the hypothesis that NadE^Gln^ and PII could physically interact, and PII proteins could somehow affect the NadE^Gln^ function (12).

The PII proteins are widespread signal transduction proteins present in a broad range of prokaryotes and in the chloroplast of eukaryotic phototrophs (12). In addition to the *glnB* gene, some organisms can encode additional PII gene paralogues. In Proteobacteria, the second PII gene is named *glnK* and is encoded in an operon along with the *amtB* gene (13). In the special case of *Azospirillum brasilense,* the *glnK*-like gene is not co-transcribed with *amtB* and is named GlnZ (11).

The PII protein structure is highly conserved and forms a compact homotrimeric barrel with an extraordinary ability to sense and integrate the levels of key metabolites such as ATP, ADP, 2-oxoglutarate (2-OG)^4^ and in certain cases l-glutamine (14). These metabolites represent critical signals of the nutritional status of the cell as they reflect the availability of energy (ATP/ADP ratio), nitrogen (glutamine acts as a signal of nitrogen availability), and the carbon/nitrogen ratio (2-OG acts as a signal of the carbon/nitrogen balance) (15).

The nucleotides ATP and ADP bind competitively to the three nucleotide-binding sites located in the clefts formed between each PII subunit (16). The three 2-OG binding sites in the PII trimer are formed only when PII is pre-occupied with MgATP. This cooperative binding of MgATP and 2-OG results in enhanced ATP affinity in the presence of 2-OG (17). Thus, in a competition between ATP and ADP, the presence of high levels of 2-OG favors the ATP binding to PII (18, 19). The interplay between the allosteric effectors 2-OG, ATP, and ADP is a conserved feature of the PII protein family (12). In response to varying levels of these allosteric effectors, PII may exist theoretically in up to 21 different structural conformations (20). Although not all of them may play a physiologic role, structural changes indeed affect the ability of PII to interact and regulate essential metabolic proteins, thereby pacing the overall cellular metabolism accordingly to nutrient availability (15).

The PII proteins also respond to the levels of glutamine. However, the mechanism of regulation by glutamine is not universal. In Proteobacteria, PII proteins are subject to a cycle of reversible uridylylation of a Tyr residue located at the apex of a solvent-exposed loop, namely T-loop. This response is mediated by the glutamine-sensitive bifunctional uridylyltransferase/removing enzyme GlnD (21). On the other hand, plants and eukaryotic algae evolved PII proteins that are regulated by glutamine because of direct allosteric binding (22). Despite the mechanism used for glutamine sensing, glutamine levels affect the PII protein function to coordinate cellular metabolism accordingly to the availability of nitrogen.

Here we show that type 2 NadE^Gln^ enzymes are negatively feedback-inhibited by physiological levels of NAD^+^, and this regulatory mechanism is conserved in distantly related bacteria. We show that PII proteins act as a dissociable regulatory subunit of dimeric NadE2^Gln^ in bacteria. Complex formation between PII and NadE2^Gln^ relieves the NadE2^Gln^ inhibition by NAD^+^, thereby acting as a switch to coordinate NAD^+^ production with nutrient availability in prokaryotes.

## Results

### Identification of the dimeric NadE^Gln^ as a novel target of the signal transduction protein GlnZ

We used Ni-NTA column loaded with N-terminal His-tagged GlnZ in an attempt to identify novel PII-binding proteins in the diazotrophic α-Proteobacterium *A. brasilense*. This ligand fishing GlnZ-affinity column was then challenged with *A. brasilense* protein extracts in the presence of MgATP. A blank nickel column, without His-GlnZ, was used as a negative control. After extensive washes with buffer containing MgATP, both columns were washed with buffer containing MgATP plus 1.5 mm 2-OG. Two consecutive fractions of 1.5 ml (fractions 1 and 2) eluted with 2-OG were collected and analyzed by label-free LC-MS/MS. The rationale of this approach is that, with the addition of 2-OG, GlnZ would assume a different conformation and, therefore, release proteins that were specifically retained in the column because of direct physical interaction with GlnZ.

The top five proteins that were specifically enriched in the fractions eluted with MgATP and 2-OG from the His-GlnZ affinity column compared with the control column were two putative uncharacterized proteins; orotate phosphoribosyltransferase, which is part of the pyrimidine biosynthetic pathway; pyruvate-phosphate dikinase regulatory protein, a bifunctional serine/threonine kinase/phosphatase that regulates pyruvate-phosphate dikinase; type 2 glutamine-dependent NAD synthetase, namely AbNadE2^Gln^, that has been studied previously by our group (Fig. S1, UniProt G8AIW8) (7).

### In vitro complex formation between purified GlnZ and A. brasilense NadE2

**Figure 2.**
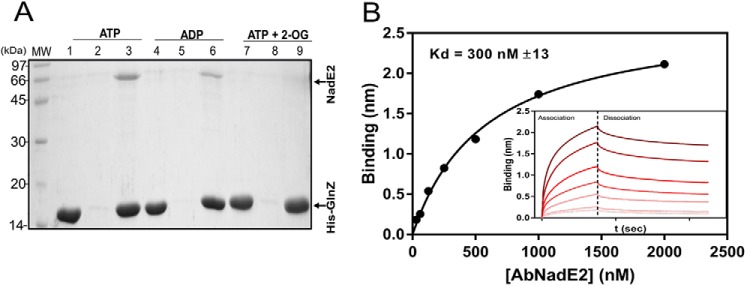
***In vitro* complex formation between AbNadE2 and GlnZ.** A, protein complex formation was assessed by pulldown using Ni^2+^ beads. Assays were performed in the presence of 5 mm MgCl_2_ and the effectors ATP, ADP, and/or 2-OG at 1 mm, as indicated. Binding reactions were conducted in 400 μl of buffer, adding purified His-GlnZ mixed with native AbNadE2. After extensive washes, bound proteins were eluted with SDS-PAGE loading buffer and analyzed by SDS-PAGE stained with Coomassie Blue. *Lanes 1*, *4*, and *7*, HisGlnZ only. *Lanes 2*, *5*, and *8*, AbNadE2 only. *Lanes 3*, *6*, and *9*, a mixture of HisGlnZ and AbNadE2. *B*, biolayer interferometry quantification of GlnZ-AbNadE2 interaction. His-GlnZ was immobilized on the Ni-NTA sensor tip in a concentration of 2 μg/ml. The sensor tip was then challenged in a solution containing the indicated AbNadE2 concentrations in the presence of 5 mm MgCl_2_ and 1 mm ATP. *Insert,* plots reporting the Δλ spectral shift in nm *versus* time in response to increasing AbNadE2 concentrations. The binding curves and *K_d_* were determined and calculated using the manufactureŕs software (FortéBio) with the determined association and dissociation rates (*k*_ON_ = 9.09 × 10^4^ m^−1^ s^−1^, *k*_OFF_ = 2.72 × 10^−2^ s^−1^).

The formation of a PII-NadE^Gln^ complex has been previously suggested by bioinformatic analysis (11). Hence, we focused our efforts to confirm the interaction between *A. brasilense* GlnZ and AbNadE2 using the purified components *in vitro*. The AbNadE2 enzyme was purified to homogeneity and tested for interaction with the N-terminal His-tagged GlnZ protein using pulldown assays. When ATP or ADP was present in the buffers, AbNadE2 was co-purified with His-GlnZ using nickel beads (Fig. 2*A*). When the pulldown assays were performed in the presence of MgATP and saturating 2-OG concentrations, no interaction between GlnZ and AbNadE2 could be detected (Fig. 2*A*).

The interaction between GlnZ and AbNadE2 was then quantitatively analyzed using Biolayer interferometry. The His-tagged GlnZ was mobilized on Ni-NTA tips and exposed to various AbNadE2 solutions in presence of different effector molecules. The binding curves obtained with increasing concentrations of AbNadE2 enabled the estimation of a *K_d_* of 300 nm in the presence of ATP and a *K_d_* of 150 nm in the presence of ADP (Fig. 2*B* and Fig. S2, respectively).

### The AbNadE2 is feedback-inhibited by NAD^+^

To determine whether the GlnZ–AbNadE2 interaction would affect the AbNadE2 activity, enzymatic assays were performed by combining the substrates l-glutamine, ATP, and NaAD. The formation of NAD^+^ by AbNadE2 was determined spectrophotometrically after its conversion to NADH by alcohol dehydrogenase, as described previously (7). Strikingly, despite complex formation, GlnZ had no effect on AbNadE2 activity using saturating or subsaturating concentrations of the AbNadE2 substrates (data not shown).

We reported previously and confirmed here that AbNadE2 is more active in a reducing environment in the presence of DTT using glutamine as N donor (Fig. S3). This feature seems to be a general characteristic of dimeric NadE2^Gln^ as we also noted this effect with orthologous enzymes from the β-Proteobacterium *Herbaspirillum seropedicae* (HsNadE2^Gln^) and from the Cyanobacteria *Synechocystis* sp. (ScNadE^Gln^) (Fig. S3*A*). On the other hand, the presence of DTT had no effect when l-glutamine was substituted by ammonium as N donor for the reaction (Fig. S3*B*). These data suggest that NadE2^Gln^ enzymes require a reducing environment for the full activity of the glutaminase domain.

**Figure 3.**
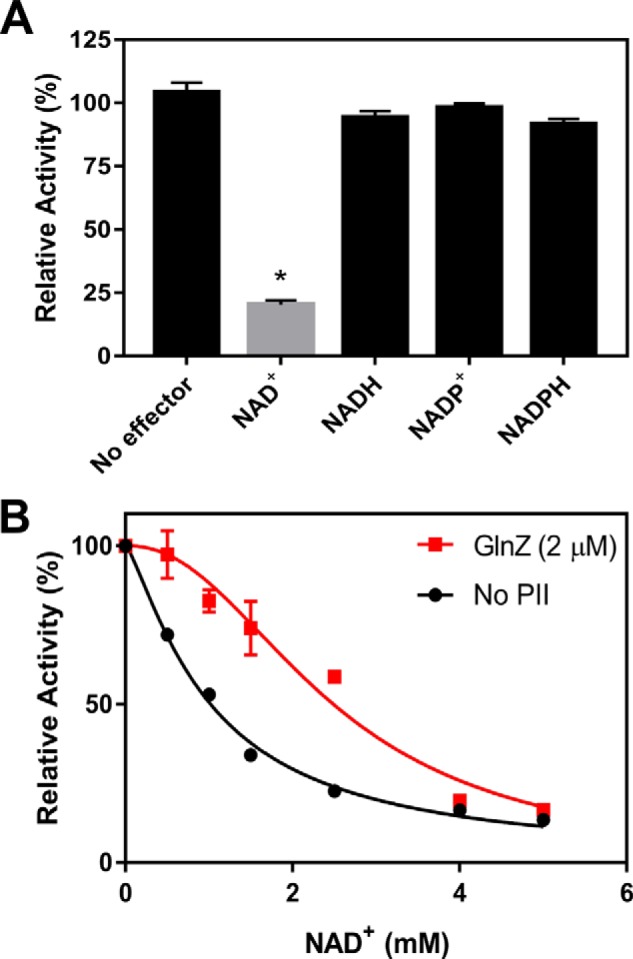
**The AbNadE2 activity is down-regulated by NAD^+^ and GlnZ relives AbNadE2 from NAD^+^ inhibition.** NadE2 discontinuous assays were performed to measure the formation of PP_i_ in the presence of 4 mm l-glutamine, 4 mm ATP, and 2 mm NaAD. Data were plotted as relative percent activity considering the reaction without NAD^+^ as 100% activity. *A*, effect of redox metabolites, when indicated 2.5 mm NAD^+^ or 1 mm of NADH, NADP^+^ or NADPH were added. An *asterisk* indicates statistically significant difference (*t* test, *p* < 0.01), *n* = 3. *B*, reactions were carried out in the presence of 100 nm NadE2Ab (monomer), GlnZ was added at 2 μm (trimer) when indicated. Reactions without GlnZ were contained 2 μm BSA. The calculated AbNadE2 NAD^+^ *K_i_* was 1 mm and 2.5 mm in the absence and presence of GlnZ, respectively. Data were analyzed and the IC_50_ were calculated by nonlinear regression in GraphPad Prism 7. S. D. from triplicate experiments are indicated by *error bars*.

Given the role of NadE in NAD^+^ homeostasis and its possible regulation by the redox status, we thought that some of the cell redox metabolites and the final products of NAD^+^ biosynthetic pathway (NAD^+^, NADH, NADP^+^, and NADPH) could play a role in the regulation of AbNadE2. Hence, AbNadE2 activity was measured by monitoring PP_i_ released in the presence of these metabolites. We found that NAD^+^ acted as a potent inhibitor of AbNadE2, diminishing the enzyme activity to ∼17%. The other tested metabolites showed no effect (Fig. 3*A*). The presence of NAD^+^ inhibited AbNadE2 in a dose-dependent hyperbolic curve with an estimated *K_i_* of 1 mm (Fig. 3*B*).

The structural similarities between NaAD and NAD^+^ (Fig. 1) led us to investigate if the presence of NAD^+^ could inhibit NadE activity by competing with the NaAD substrate (competitive inhibition). The type of inhibition can be distinguished by the kinetic constants, which were, therefore, determined. The presence of NAD^+^ did not affect the AbNadE2 NaAD *K_m_*, but instead, this metabolite altered AbNadE2 *V*_max_ (from 0.09 μmol s^−1^ to 0.04 μmol s^−1^ in the presence of NAD^+^) (Fig. S4*A*). The l-glutamine *K_m_* was also unaffected in the presence of 1 mm NAD^+^ (Fig. S4*B*). These data indicate the presence of an allosteric NAD^+^ inhibitory binding site on AbNadE2.

### The feedback inhibition of AbNadE2 by NAD^+^ is relieved by GlnZ

The presence of GlnZ did not affect AbNadE2 activity in the presence or absence of 5 mm NAD^+^ (Fig. 3*B*). However, GlnZ was able to partially relieve the inhibitory effect on AbNadE2 activity when NAD^+^ was present under physiological relevant levels (between 0.5 and 2.5 mm). The AbNadE2 NAD^+^ inhibition curve changed from hyperbolic to sigmoidal in the presence of GlnZ (Fig. 3*B*), increasing the IC_50_ from 1 mm in the absence of GlnZ to 2.5 mm in its presence. The AbNadE2 NAD^+^ IC_50_ in the presence of GlnZ is near the reported intracellular NAD^+^ concentration in *Escherichia coli* of 2.6 mm (23).

**Figure 4.**
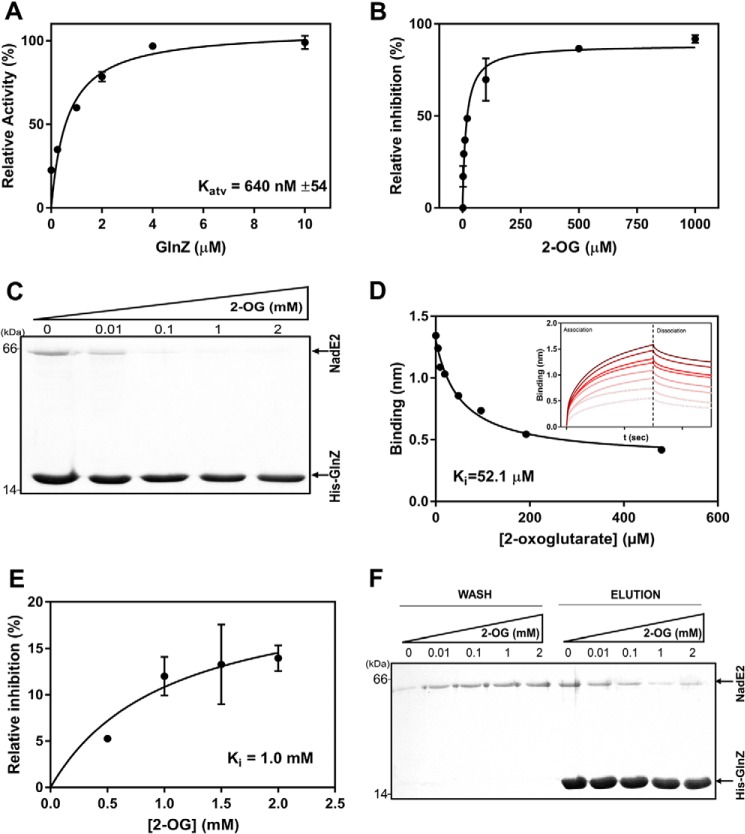
**Effect of 2-oxoglutarate in the dissociation of GlnZ-NadE2Ab complex and enzymatic activity of AbNadE2.** NadE2 discontinuous assays were performed to measure the formation of PP_i_ in the presence of 4 mm l-glutamine, 4 mm ATP, and 2 mm NaAD. Data were plotted as relative percent activity considering the reaction without NAD^+^ as 100% activity. *A*, reactions contained 100 nm NadE2Ab and 2.5 mm of NAD^+^. GlnZ was added at 0.25, 1, 2, 4, and 10 μm (trimer). The estimated *K_d_* for the AbNadE2-GlnZ complex was 640 nm and was calculated using nonlinear regression in GraphPad Prism 7. *B*, the activity of AbNadE2 in the presence of GlnZ is regulated by the levels of 2-OG. Data were plotted as percent inhibition relative to the AbNadE2 activity in the presence of NAD^+^, which was considered as 100% inhibition. Assays were performed in the presence of 2.5 mm NAD^+^, 2 μm GlnZ, and increasing 2-OG concentrations. The estimated IC_50_ (*K_i_*) of 2-OG was 15.5 μm. *C*, the formation of the GlnZ-AbNadE2 complex was assessed by pulldown using Ni^2+^ beads. Reactions were performed in the presence of MgCl_2_ 5 mm, 1 mm ATP, and increasing concentrations of 2-OG as indicated. Binding reactions were conducted in 400 μl of buffer, adding purified His-GlnZ and untagged AbNadE2. After extensive washes, bound proteins were eluted with SDS-PAGE loading buffer and analyzed by SDS-PAGE stained with Coomassie Blue. *D*, biolayer interferometry analysis of the GlnZ-AbNadE2 complex. The His-GlnZ was immobilized in the Ni-NTA sensor tip. The tip was then steeped in a solution containing 400 nm AbNadE2, MgCl_2_ 5 mm, 1 mm ATP, and different concentrations of 2-OG. *Insert*, plots reporting the Δλ spectral shift in nm *versus* time under different 2-OG concentrations. The binding curves and *K_i_* were determined using the manufactureŕs software (FortéBio). *E*, curve of AbNadE2 relative inhibition in response to increasing 2-OG concentrations. The AbNadE2 inhibition in the presence of 5 mm NAD^+^ is considered 100% inhibition. Reactions were carried in the presence of 2.5 mm NAD^+^ and 2 μm GlnZ. The GlnZ and NadE2 proteins were preincubated in MgATP before the addition of 2-OG and the start of the reaction. Hence, this assay reflects the ability of 2-OG to dissociate a pre-formed GlnZ-AbNadE2 complex. The estimated *K_i_* for 2-OG was 1 mm and calculated nonlinear regression in GraphPad Prism 7. *F*, the HisGlnZ-NadE2 complex was immobilized on Ni^2+^ beads in the presence of 1 mm ATP. Beads were washed (*Wash*) with buffer containing ATP (1 mm) and the indicated 2-OG concentrations. Bound proteins were eluted in SDS-PAGE sample buffer (*Elution*). The samples were applied to SDS-PAGE 15%, and the gels were stained with Coomassie Blue.

When NAD^+^ was kept constant at the physiologically relevant concentration of 2.5 mm, GlnZ was able to activate AbNadE2 in a hyperbolic GlnZ dose-dependent curve (Fig. 4*A*). The estimated *K*_act_ was 640 nm, which is in the same range as the *K_d_* for the GlnZ-AbNadE2 complex estimated using Biolayer interferometry (Fig. 2*B*).

Given the negative effect of 2-OG on the formation of the GlnZ-AbNadE2 complex (Fig. 2*A*), we thought whether 2-OG could impair the ability of GlnZ to relive AbNadE2 NAD^+^ inhibition. When AbNadE2 activity was measured in the presence of 2.5 mm NAD^+^ and GlnZ was first equilibrated in buffer containing 2-OG before combining with AbNadE2, 2-OG impaired the ability of GlnZ to relive AbNadE2 NAD^+^ inhibition in a 2-OG–dependent manner (Fig. 4*B*). The response to 2-OG was hyperbolic with a *K_i_* for 2-OG of 15 μm (Fig. 4*B*). The *K_i_* for 2-OG is in the same range as the *K_i_* for 2-OG–dependent inhibition of GlnZ-AbNadE2 complex formation, as determined by pulldown or Biolayer interferometry (Fig. 4, *C* and *D*).

When AbNadE2 activity was measured by mixing GlnZ and AbNadE2 before the addition of 2-OG, a different response to increasing 2-OG levels was observed. The effect of 2-OG was much less pronounced, with 2-OG showing a *K_i_* of 1 mm (compare Fig. 4, *B* and *E*, and Fig. 4, *C* and *F*). Note that although the experiments depicted in Fig. 4*B* reflect the ability of 2-OG to inhibit GlnZ-AbNadE2 complex formation, the assay in Fig. 4*F* reflects the ability of 2-OG to dissociate a pre-formed GlnZ-AbNadE2 complex. Pulldown assays indicated that concentrations of 2-OG up to 2 mm were not enough to fully dissociate a pre-formed GlnZ-AbNadE2 complex (Fig. 4*E*). On the other hand, 2 mm 2-OG completely abolished GlnZ-AbNadE2 complex formation (Fig. 4*C*). These data imply that once the GlnZ-AbNadE2 complex is formed, 2-OG is much less effective to impair the interaction between these proteins and, hence, to relieve AbNadE2 NAD^+^ inhibition.

### Effects of GlnZ uridylylation and the GlnZ T-loop on AbNadE2 activity

**Figure 5.**
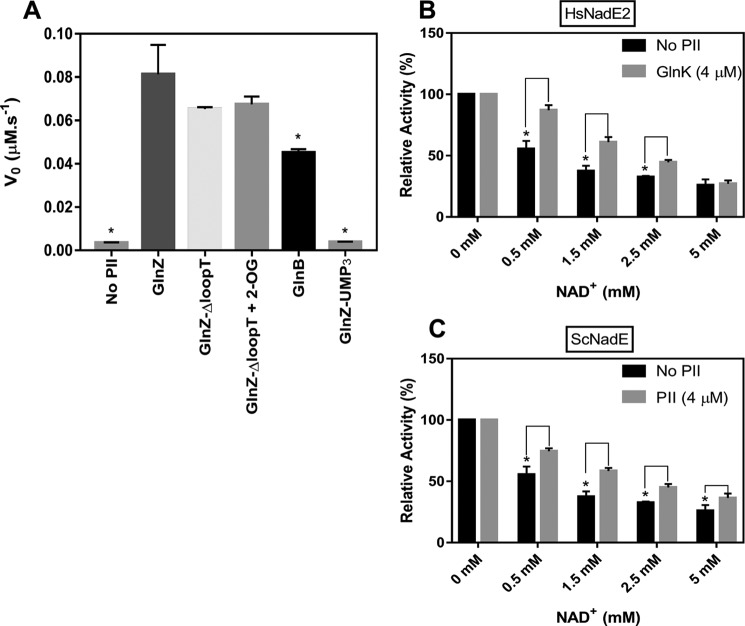
**Effect of PII protein on NadE2 activity depends on the PII variant and is conserved in different bacteria.** *A*, the activity of AbNadE2 was measured in the presence of MgCl_2_ 5 mm, 1 mm ATP, 2.5 mm NAD^+^, and the indicated PII protein (GlnZ, GlnZΔloopT, GlnB, and GlnZ-UMP_3_) at 2 μm concentration (trimer), 1.5 mm 2-OG was added when indicated. Treatment without PII was carried with 2 μm of BSA. Activity measured following PP_I_ production. An *asterisk* indicates the statistically significant difference compared with GlnZ (*t* test, *p* < 0.01), *n* = 3. *B* and *C*, NadE2 activity data were plotted as relative percent activity considering the reaction without NAD^+^ as 100% activity. All reactions were carried out in the presence of 100 nm HsNadE2 (*B*) or ScNadE2 (*C*), 4 mm ATP, 2 mm NaAD, and 4 mm l-glutamine. When indicated, the corresponding PII protein was added at 2 μm (trimer). Curves without PII were carried out using 2 μm BSA. Activity measured following PP_I_ with EnzChek Pyrophosphate Assay Kit. An *asterisk* indicates a statistically significant difference compared (*t* test, *p* < 0.01); *n* = 3.

Under nitrogen-limiting conditions, GlnZ is predominantly found in fully uridylylated form *in vivo* (24). Using *in vitro* uridylylated GlnZ protein, we could show that, in contrast to nonuridylylated GlnZ, GlnZ-UMP_3_ was not able to relieve AbNadE2 NAD^+^ inhibition (Fig. 5*A*). Furthermore, GlnZ-UMP_3_ could not interact with AbNadE2, as indicated by pulldown assays (data not shown). Hence, the uridylylation of GlnZ abrogates its ability to interact with AbNadE2 and to relieve the NAD^+^ inhibition.

Most of the known interactions between PII proteins and their target proteins occur via the T-loop of the PII protein. Indeed, this is the region that suffers the most prominent conformational change upon 2-OG binding (17). We tested the ability of a GlnZ variant carrying a deletion of the T-loop (GlnZ ΔQ42-S54) to interact with AbNadE2. The delta T-loop variant was still able to interact with GlnZ, but the complex formation was unresponsive to 2-OG (Fig. S5*A*). In agreement with the pulldown data, the GlnZ variant ΔQ42-S54 was able to relieve the AbNadE2 NAD^+^ inhibition despite the presence of 2-OG (Fig. 5*A*).

*A. brasilense* encodes a second PII paralogue, namely GlnB, which is 67% identical to GlnZ at the amino acid sequence (25). To determine whether GlnB could also interact with AbNadE2, pulldown assays were performed using His-GlnB or His-GlnZ as a bait in the presence of ATP. Even though AbNadE2 could be co-purified with His-GlnB, the amount of AbNadE2 protein recovered was higher using His-GlnZ as bait (Fig. S5*B*). In agreement, GlnB was much less effective than GlnZ in relieving the AbNadE2 NAD^+^ inhibition (Fig. 5*A*). These data suggest that both *A. brasilense* PII paralogues can interact with AbNadE2, but a more stable complex is apparently formed with the GlnZ paralogue.

### The regulatory interplay between NAD^+^ and PII proteins is conserved among bacterial dimeric glutamine–dependent NadE^Gln^

To determine whether regulatory interplay between NAD^+^ and PII proteins is conserved among NadE2^Gln^ in bacteria, the NadE2^Gln^ enzymes from the β-Proteobacterium *H. seropedicae* and the Cyanobacteria *Synechocystis* sp. were purified to homogeneity and tested for enzymatic activity in the presence of NAD^+^ and the GlnZ/GlnB orthologue from the respective organism. In both cases, NadE2^Gln^ was inhibited by NAD^+^ in a dose-dependent manner within the physiological range (Fig. 5, *B* and *C*). For both organisms, the presence of PII relieved NadE2^Gln^ NAD^+^ inhibition, although the PII effect was less pronounced in the case of *Synechocystis* sp NadE2 (Fig. 5*B*). These data suggest that both the inhibition by NAD^+^ and regulation by PII are conserved mechanisms to regulate NadE2^Gln^ in bacteria.

### Bioinformatic analysis of the PII-nadE gene islands

Inspection of the PIRSF database (26) identified 19,848 sequences homologous and homeomorphic to AbNadE2 (*i.e.* containing similar domain architecture, a glutaminase domain fused to NAD synthetase domain, PIRSF accession 006630). These sequences are distributed in 1772 eukaryotes, 17,578 bacteria, and 222 Archaea. Hence, glutamine-dependent NadE enzymes are widespread throughout nature.

The conservation of gene order can be used as a fingerprint of proteins that physically interact (10). We have set a bioinformatic approach to identify *PII-nadE* genetic islands in prokaryotes. A total of 1300 nonredundant *PII-nadE* islands were identified (Table S1). Most of the *PII-nadE* islands identified belonged to β- and γ-Proteobacteria, 834 and 436 counts, respectively (Table S1). *PII-nadE* islands were identified in distantly related prokaryotes such as the bacteria phylum Firmicutes, Nitrospirae, Actinobacteria, Deinococcus-Thermus, Ignavibacteriae; and the Archaea phylum Euryarchaeota, Crenarchaeota (Table S1). Interestingly, in *A. brasilense* and *Synechocystis* sp., the PII gene is not linked *nadE*; PII nevertheless regulated the NadE^Gln^ function (Figs. 3*B* and 5*C*). Hence, NadE^Gln^ regulation by PII can occur despite the absence of a genetic link of respective genes in these cases. The bioinformatic analysis reinforces that the formation of the PII-NadE complex may be a conserved feature in bacteria and may also be present in Archaea.

**Figure 6.**
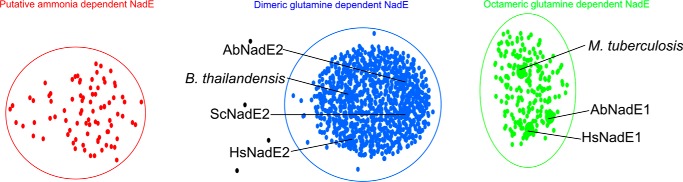
**Similarity based grouping of NadE orthologues identified in the PII-NadE genomic islands.** All the NadE sequences retrieved from the identified PII-NadE islands were compared using all-against-all BLAST searches using CLANS. Each protein sequence is displayed as a *dot* and the pairwise similarities are presented in a *graph* according to the similarity distances. Three major clusters were formed. The sequences of previously characterized dimeric and octameric glutamine-dependent NadE were included as function group guide in the analysis and are indicated. The *green* cluster included the octameric NadE1^Gln^ from *Mycobacterium tuberculosis*, NadE1^Gln^ from *Herbaspirillum seropedicae* (HsNadE1), and NadE1^Gln^ from *Azospirillum brasilense* (AbNadE1). The *blue cluster* included the dimeric glutamine-dependent NadE enzymes from *H. seropedicae* (HsNadE2), NadE2^Gln^ from *A. brasilense* (AbNadE2), NadE^Gln^ from *Burkholderia thailandensis*, and *Synechocystis* sp. (ScNadE2). The *red cluster* includes NadE sequences lacking the N-terminal glutaminase domain and thus were considered putative ammonia-dependent NadE.

The NadE sequences identified within the *PII-nadE* islands were separated into three major groups using CLANS (Fig. 6). Most of the retrieved NadE sequences belonged to dimeric NadE^Gln^. Interestingly, a significant number of octameric glutamine-dependent NadE^Gln^ synthetases were also retrieved in this analysis, along with few members of ammonium-dependent NadE^NH3^ enzymes (Fig. 6). These data suggest that regulation of NAD^+^ biosynthesis by PII may be even more widespread in nature and not only restricted to dimeric type 2 NadE^Gln^.

## Discussion

The biosynthesis of NAD^+^ is a vital metabolic pathway in bacteria such that the suppression of the enzyme catalyzing the last step of NAD biosynthesis, NadE, resulted in bactericidal effects (27) because of nicotinamide nucleotides being essential cofactors in redox catalysis, and the total concentration of these molecules must cover their requirement for cell metabolism. In addition to this well-established role, NAD^+^ also serves as a substrate for sirtuins (lysine deacetylases), ADP-ribosylating enzymes, DNA ligase, and NAD^+^ kinase and may act as a signaling metabolite (1, 2). For these reasons, the production of NAD^+^ must be coordinated with its consumption and cell growth to keep the nicotinamide nucleotide levels homeostatic.

Here we show that the glutamine-dependent dimeric NadE2 from distantly related bacteria such as from *A. brasilense* (α-Proteobacteria), *H. seropedicae* (β-Proteobacteria), and *Synechocystis* sp (Cyanobacteria) are negative feedback–inhibited by physiologically relevant concentrations of NAD^+^ (Figs. 3*B* and 5, *B* and *C*). We speculate that bacterial NadE2^Gln^ may be universally inhibited by NAD^+^. Kinetic analysis with AbNadE2 showed that NAD^+^ does not compete with the binding of substrate NaAD but reduces the enzymes *V*_max_ (Fig. S4). This clearly indicates that NadE2^Gln^ is feedback-inhibited through an allosteric inhibitory binding site for NAD^+^.

Initially, we used a PII protein affinity column to successfully identify AbNadE2 as a novel target of the PII protein GlnZ in the diazotrophic α-Proteobacterium *A. brasilense* (Fig. 2*A* and Fig. S1). The presence of GlnZ changed the response of AbNadE2 to its feedback inhibitor NAD^+^. Instead of a hyperbolic inhibition curve with a *K_i_* of 1 mm, the GlnZ-AbNadE2 complex displayed a sigmoidal inhibition curve with an IC_50_ of 2.5 mm. Thereby, GlnZ relived the AbNadE2 NAD^+^ inhibitory effect in particular under physiologically relevant NAD^+^ concentrations (Fig. 3*B*). The sigmoidal dose-response curve indicates the cooperativity of the NAD^+^ allosteric binding sites in the GlnZ-AbNadE2 complex (Hill coefficient 2.2). The presence of high 2-OG levels or GlnZ uridylylation impaired the formation of the AbNadE2-GlnZ complex, thereby preventing GlnZ to revert AbNadE2 NAD^+^ inhibition (Figs. 4*D* and 5*A*).

**Figure 7.**
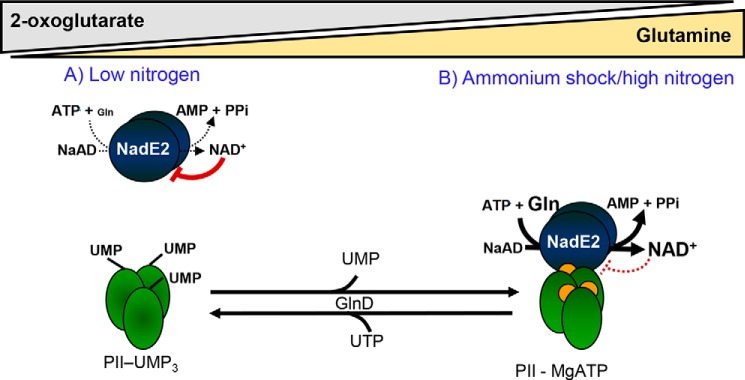
**The PII proteins act as a dissociable regulatory subunit of NadE2^Gln^ to pace NAD^+^ production accordingly to the availability of l-glutamine and 2-OG.** *A*, when nitrogen is limited, cell growth is arrested, the intracellular levels of 2-OG are high, and glutamine are low. PII is fully uridylylated and does not interact with NadE2, resulting in strong feedback negative inhibition of NadE2 by NAD^+^. *B*, when ammonium becomes available, glutamine rises and 2-OG drops, PII is deuridylylated (reaction catalyzed by the GlnD enzyme in response to high glutamine) and bound to MgATP and/or ADP (*orange dots*). Under this condition, PII interacts with NadE2 relieving NAD^+^ inhibition and thus allowing higher NAD^+^ production rates to feed the demands of active cell growth.

From these data, we propose a model where the PII protein acts as a dissociable regulatory subunit of NadE2^Gln^ to regulate NAD biosynthesis according to the metabolic state sensed by GlnZ (Fig. 7). Under low nitrogen availability, glutamine levels are low, and 2-OG levels are high (23, 28), PII (GlnZ) is uridylylated, resulting in diminished flux through NadE2 because of NAD^+^ feedback inhibition and low availability of the l-glutamine substrate (Fig. 7*A*). Upon an ammonium shock, glutamine levels rise (7), and PII is rapidly deuridylylated (24), 2-OG levels reduce because of its use in ammonium assimilatory reactions (15, 29). These conditions favor the formation of the NadE2-PII complex, thereby relieving the NAD^+^ inhibition over NadE2, allowing increased flux through NadE2 (Fig. 7*B*). This is of particular importance in the nitrogen-fixing bacterium *A. brasilense,* which possesses a mechanism to inactivate the abundant enzyme nitrogenase by ADP ribosylation as an immediate response to ammonium shock (30). The nitrogenase ADP-ribosyl-transferase DraT uses NAD^+^ as a substrate and is activated upon interaction with the PII protein GlnB (31, 32) precisely under the conditions where GlnZ activates NadE2 (Fig. 7). Hence, the concerted activation of the NAD^+^ consuming enzyme, DraT, with the NAD^+^ producing enzyme, NadE2, by the paralogue PII proteins, would ensure NAD^+^ homeostasis during the enzymatic consumption of this cofactor.

Our *in vitro* data indicate that once the PII-NadE2 complex is formed, higher levels of 2-OG are needed to dissociate the complex compared with the 2-OG levels required to inhibit complex formation (Fig. 4, *E* and *F*). Apparently, the PII affinity for 2-OG binding is reduced within the PII-NadE2 complex compared with free PII. This may result in a “memory effect” such that once the GlnZ-AbNadE2 complex is formed, it would become resistant to small and transient fluctuations in 2-OG concentrations. That the affinity of PII proteins toward the effector molecules is differentially modulated through complex formation with various targets has already been described at a structural level for cyanobacterial PII protein (14, 34).

The action of PII to tune down feedback inhibition of NadE2 by NAD^+^ is conserved in distantly related bacterial such as *H. seropedicae* (β-Proteobacteria) or *Synechocystis* sp (Cyanobacteria) (Fig. 5, *B* and *C*). Hence, the model depicted in Fig. 7 may be applied for other prokaryotes, as well. The conservation of gene order is a fingerprint of proteins that physically interact (10). The fact that the *PII-nadE* pair is conserved in widely distant prokaryotes of bacterial and archaeal origin (Table S1) suggests that the PII-NadE interaction may be widespread in prokaryotes. This is in analogy to the conserved *PII-amtB* genetic pair, whose protein products were shown to physically interact in a broad range of prokaryotes, including bacteria and Archaea (12, 35). Our gene neighborhood analysis also suggests that other types of NAD synthetases, NadE1^Gln^ and NadE^NH3^, could be regulated by interaction with PII (Fig. 6). This hypothesis is currently under investigation.

The unique ability of the PII protein to sense and integrate the levels of essential metabolites such as ATP, ADP, 2-OG, and glutamine was capitalized by nature in such a way that PII regulates a range of key metabolic enzymes, transporters, and transcriptional regulators by direct protein– interactions (12). In addition to the well-established control of the nitrogen metabolism, recent data showed that PII regulates other key metabolic pathways, such as those involved in fatty acid production (36, 37). The identification of NadE2^Gln^ as a novel PII target protein supports the notion that PII may function as a central orchestration unit of metabolism to tune the flux trough key metabolic pathways according to the metabolic state determined by the availability of nutrients and energy supply.

When nutrients and energy are plentiful, microbes achieve maximum growth rates, and under these conditions, the production of NAD^+^ and its derivatives (NADH, NADP, and NADPH) must increase to keep pace with cell growth, providing enough cofactors for daughter cells. Indeed, single-cell fluorescence measurements indicate a peak of NADH accumulation during the progress to cell division in *E. coli* (38). As the new findings pointed to regulation through NAD^+^ levels although NADH did not affect NadE2^Gln^, we speculate that oscillations in the NADH/NAD^+^ ratio may play a role in the NAD^+^ biosynthesis and thus in regulation through PII transduction proteins. The mechanism responsible for coordinating NAD^+^ production with nutrient availability has remained elusive. The PII proteins, with their extraordinary sensory properties, are ideally suited to fulfill this role.

The NAD^+^ levels play important signaling roles in eukaryotes by regulating processes such as DNA repair, cell cycle progression, gene expression, and calcium signaling (6, 39). Impaired biosynthesis and increased NAD^+^ consumption are associated with aging and pathologies in mammals (39). The use of NAD^+^ as a substrate for a range of enzyme prokaryotic enzymes, including those involved in RNA modification (40), sirtuins (41), and ADP-ribosylation (42), suggest that NAD^+^ also play important regulatory roles in prokaryotes. The key metabolic function makes NAD^+^ well-suited for signaling. The interplay between the levels of ATP, ADP, 2-OG, glutamine (sensed by PII), and NAD^+^ (sensed by NadE2) through the formation of the PII-NadE2 complex, constitutes a novel metabolic hub that may act to balance the levels of core cellular metabolites.

## Experimental procedures

### Ligand fishing His-GlnZ affinity chromatography

To prepare the *A. brasilense* protein extract, 400 ml of the *A. brasilense* 2812 strain was cultivated on NFbHP to an *A*_600 nm_ of 2. Cells were collected by centrifugation, resuspended in 20 ml of buffer containing 50 mm Tris-HCl, pH 8, 100 mm KCl, 20 mm imidazole, 5 mm MgCl_2_, 1 mm ATP and disrupted by sonication. The soluble fraction (after centrifugation at 30,000 × *g*, 4 °C for 30 min) was divided into two aliquots, one was the control fraction and the other the GlnZ affinity ligand fishing.

For the preparation of the His-GlnZ extract, 300 ml of *E. coli* BL21(DE3) carrying the pMAS3 plasmid that expresses the *A. brasilense* His-GlnZ protein was cultured in LB medium at 37 °C until *A*_600 nm_ of 0.5. Then, 0.3 mm IPTG was added, and after 4 h, cells were harvested by centrifugation and resuspended in buffer containing 50 mm Tris-HCl, pH 8, 100 mm KCl, and 20 mm imidazole. Cells were disrupted by sonication and the soluble fraction obtained after centrifugation (30,000 × *g* at 4 °C for 30 min).

For interaction assay, two Hi-trap chelating columns (1 ml) (GE Healthcare) charged with Ni^2+^ were used. The first one was equilibrated with buffer containing 50 mm Tris-HCl, pH 8, 100 mm KCl, and 20 mm imidazole and loaded with His-GlnZ extract, washed with 20 ml of buffer containing 50 mm Tris-HCl, pH 8, 100 mm KCl, and 50 mm of imidazole to remove unspecific bound proteins and equilibrated with 5 ml of buffer containing 50 mm Tris-HCl, pH 8, 100 mm KCl, 50 mm imidazole, 5 mm MgCl_2_, 1 mm ATP. This column is prepared to bind the putative targets of GlnZ. The second column was equilibrated with 5 ml of buffer containing 50 mm Tris-HCl, pH 8, 100 mm KCl, 5 mm MgCl_2_, 1 mm ATP, and 50 mm imidazole. This column is a control for the unspecific binding of proteins to the column resin, thus, providing a control fraction of proteins eluted in the ligand fishing assay but not bound to GlnZ. Both columns were loaded with *A. brasilense* protein extract and washed with 20 ml of buffer containing 50 mm Tris-HCl, pH 8, 100 mm KCl, 5 mm MgCl_2_, 1 mm ATP, and 50 mm imidazole.

Proteins bound to both columns were eluted with 3 ml of buffer containing 1.5 mm 2-OG and 50 mm Tris-HCl, pH 8, 100 mm KCl, 5 mm MgCl_2_, 1 mm ATP, 50 mm imidazole. Two fractions of 1.5 ml (namely Fractions 1 and 2) were collected from the control and His-GlnZ columns and analyzed by label-free quantitative LC-MS/MS as described previously (43). Proteins were identified using a UniProt *A. brasiliense* database from June 2012 (8122 sequence entries). The log enrichment ratio of each protein in the His-GlnZ column/control column in each fraction was plotted using GraphPad Prism 7 (Fig. S1).

### Construction of the plasmid expressing NAD synthetase from Synechocystis sp, GlnD, and GlnZΔTloop from A. brasilense

The sequence of the slr1691 gene from *Synochocystis* sp. strain PCC 6803, encoding dimeric NadE2^Gln^, was retrieved from CyanoBase (44). Amplification of the gene by PCR was performed using *Synochocystis* genomic DNA as a template with the forward primer 5′-ATCACCATCACCATCACGATTACGATATCCCAACTAGTGAAAACCTGTATTTTCAGGGCGCTAGCCATATGTTTACCATTGCCCTTGCCCAGCTTAATC and the reverse primer 5′-CAGCAAAAAACCCCTCAAGACCCGTTTAGAGGCCCCAAGGGGTTATGCTAGTTATTGCTCAGCGGCCGCGGATCTTAACTGCCTTGGGGATGGAAAG.

The *glnD* sequence from *A. brasilense* was retrieved from NCBI database. The amplification by PCR was performed using *A. brasilense* genomic DNA as a template with the forward primer 5′-CTAGTGAAAACCTGTATTTTCAGGGCGCTAGCCATATGCTCTCCACCCGCGCCGCCTCCGCCGACGCGTCCGACGCCAAGGACGCCGGCACAGCCAACATCCCCAACAAG and the reverse primer 5′-CCAAGGGGTTATGCTAGTTATTGCTCAGCGGCCGCGGATCCCTCATGCGGACGGATCGGCGAGCGCGTGCAGCAGCCGCTCGCGGATCTGGGCCAGCTTGTTC. Both amplified fragments were Gibson assembled into the pTEV5 vector previously cut with NdeI and BamHI (New England Biolabs).

The gene *glnZ*Δ*Tloop* was obtained from pMSA4Δ42-54 (45) and subcloned into pET28a. The pMSA4Δ42-54 plasmid was cut with NdeI and BamHI (New England Biolabs). The resulting fragments were separated by 1% agarose gel electrophoresis and the fragment extracted using Monarch DNA Gel Extraction Kit. Gene fragment was then cloned into pET28a using NdeI/BamHI sites and transformed into DH10B cells. The kanamycin-resistant selected clone was checked by restriction pattern and it was named pGlnZΔ42-54.

The obtained plasmids (pASnadESc, pASGlnDAb, and pGlnZΔ42–54) were sequenced to confirm the integrity of the inserted genes (Table S2) (24, 48–53).

### Protein expression and purification

Recombinant protein expression was performed in *E. coli* Lemo21 (DE3) cells (New England Biolabs) in LB medium. The TEV protease was overexpressed in *E. coli* Rosetta (DE) pLysS (Novagen). Typically, *E. coli* strains carrying the expression plasmids (Table S2) (24, 48–53) were grown to an *A*_600 nm_ of 0.7 at 37 °C. Protein expression was induced by adding 0.5 mm isopropyl β-d-thiogalactopyranoside and the cell culture was incubated for 12 h at 120 rpm at 20 °C.

For purification of His-tagged PII proteins (His-GlnZ, His-AbGlnB, His-GlnZ ΔTloop (ΔQ42-S54), His-HsGlnK, or His-ScPII), cell pellets were resuspended in sonication buffer (50 mm Tris-HCl, pH 8.0, 100 mm KCl, 20 mm imidazole) and cells were disrupted by sonication (two times for 4 min, output 5 at 50% duty cycle in a Bronson sonifier), followed by centrifugation at 30,000 × *g* for 30 min to remove cell debris and insoluble material. The soluble fraction (approximately 40 ml) was loaded onto a 1-ml HisTrap HP Ni-NTA column (GE Healthcare). The column was washed with 10 ml (50 mm Tris-HCl, pH 8.0, 100 mm KCl, 50 mm imidazole) and proteins were eluted using a linear gradient of imidazole (100 to 500 mm) in the same buffer. Eluted fractions were analyzed by 15% SDS-PAGE, and the fractions containing the protein of interest were pooled and dialyzed in storage buffer (50 mm Tris-HCl, pH 8.0, 100 mm KCl, 10% glycerol). For purification of His-tagged NAD synthetase and GlnD proteins (HsNadE2, ScNadE2, and AbGlnD), the same procedure was applied with the exception that the buffers contained 100 mm NaCl instead of KCl.

To obtain untagged HsNadE2, the purified His-tagged protein was submitted to a thrombin cleavage using Thrombin CleanCleave Kit accordingly to manufacturer's instructions (Sigma-Aldrich). TEV protease was purified as described previously (46). The His-tagged ScNadE2 had its N-terminal tag removed with the TEV protease, as described previously for other NAD synthetases (7). Native AbNadE2, GlnB, and GlnZ from *A. brasilense* were purified as described previously (6, 13). The GlnZ-UMP_3_ protein was obtained by *in vitro* uridylylation of GlnZ using purified *A. brasilense* GlnD as described (47). All the proteins were stored in small aliquots at −80 °C.

### NAD synthetase activity assays

Reactions were performed at 30 °C in 50 mm Tris-HCl, pH 8, 50 mm KCl, 10 mm MgCl_2_, and indicated substrate concentrations. NadE^Gln^ activity was determined by coupling the production of NAD^+^ to the NADH-forming oxidation of ethanol using alcohol dehydrogenase, and photometric detection NADH (*A*_340 nm_). For enzyme assays, in which NAD^+^, NADH, NADP^+^, and NADPH were tested as potential effectors of NadE^Gln^, the enzyme activity was measured using a discontinuous assay over the linear phase of the reaction. Reactions were started by the addition of l-glutamine and stopped at different times on ice by adding 100 mm EDTA. The pyrophosphate product was detected using the EnzChek Pyrophosphate Assay Kit (Thermo Fisher). All the assays were performed in triplicate using a Spark microplate reader (Tecan). The initial velocity data were fitted in the equation indicated in each figure using GraphPad Prism software package.

### In vitro protein complex analysis

*In vitro* protein complex formation was assayed using His-Magnetic beads (Promega). All reactions were conducted in interaction buffer containing 50 mm Tris-HCl, pH 8, 100 mm NaCl, 5 mm MgCl_2_, 0.05% Tween 20 (v/v), 10% glycerol (v/v), 20 mm imidazole in the presence or absence of effectors (ATP, ADP, and/or 2-OG) as indicated in each experiment. Five microliters of beads were equilibrated in 200 μl of interaction buffer. The beads were recovered and resuspended in 400 μl of buffer followed by the addition of 20 μg of His-PII and 40 μg of untagged NadE protein, in this order. After 5 min at room temperature with gentle mixing, the beads were washed three times with 200 μl of interaction buffer. Bound proteins were eluted by boiling the beads in SDS-PAGE sample buffer. Proteins were analyzed by Coomassie Blue–stained 15% SDS-PAGE.

### Biolayer interferometry assays

To quantify the kinetic parameters of the PII-NadE complex an Octet K2 Bio-layer Interferometry System (FortéBio) was used. The purified proteins His-GlnZ and untagged AbNadE2 were diluted in the interaction buffer (50 mm Tris-HCl, pH 7, 50 mm KCl, 10 mm MgCl_2_, 2 mm ATP or ADP, 2.5 mm NAD^+^, and 1 mg/ml BSA). The Ni-NTA biosensor was first dipped into a solution of 2 μg/ml of His_6_-tagged GlnZ for 150 s until a binding signal of ∼2 nm was obtained (Loading). The sensor was briefly washed in binding buffer and then transferred to the analyte solution containing AbNadE2 at different concentrations for 150 s to record the association curve. Finally, the sensor was dipped into the interaction buffer for 300 s to monitor the dissociation. In parallel, a reference sensor was subjected to the same procedure, except that no His-tagged protein was bound, to determine the background of unspecific analyte binding. In a series of binding assays, AbNadE2 concentrations of 30 to 2000 nm (dimer concentration) were used to determine the *K_d_* of the GlnZ-AbNadE2 protein complex.

To determine the inhibition constant (*K_i_*) for 2-oxoglutarate on GlnZ-NadE complex formation, interaction assays using 2 μg/ml of 6xHis-tagged GlnZ and 400 nm AbNadE2 solutions were performed in the presence of different concentrations of 2-oxoglutarate (0 to 480 μm). Therefore, the Ni-NTA biosensor was loaded with His-GlnZ solution and then transferred into the AbNadE2 solution to measure the association. In a series of experiments, increasing concentrations of 2-OG were added to the interaction buffer to determine the inhibitory effect on association. The experiments were carried out in duplicate and analyzed with the Octet Data Analysis software using Savitzky-Golay filtering. The fitting of the curve was done with a 2:1 heterogeneous ligand model. Curves were then plotted in GraphPad Prism7 software.

### Redox titrations of NadE

To determine the dependence of NadE activity on the redox environment, continuous enzymatic assays were performed in the presence of saturating concentrations of 2 mm l-glutamine or 10 mm ammonium chloride. When indicated, 10 mm of freshly diluted DTT was added 10 min prior to the start of the reactions. A redox titration using different ratios of DTT and DTT_OXI_ (trans-4,5-dihydroxy-1,2-dithiane) at 10 mm total concentration was performed. The reaction buffer consisted of 50 mm Tris-HCl, pH 8, 50 mm KCl, 10 mm MgCl_2_, 2 mm ATP, and 0.2 μm of the indicated enzyme (AbNadE2, HsNadE2, and ScNadE2, monomer concentration).

### Bioinformatic analysis of PII-NadE genomic islands

Metadata of genome sequences from Archaea and Bacteria available in the NCBI Assembly database were obtained in the following addresses: ftp://ftp.ncbi.nlm.nih.gov/genomes/refseq/bacteria/assembly_summary.txt and ftp://ftp.ncbi.nlm.nih.gov/genomes/refseq/archaea/assembly_summary.txt. (Accessed 13 July, 2019) Only one genome sequence per species (or unknown species isolate) was downloaded to prevent data redundancy, giving priority to sequences obtained from type-strains and to those that represented complete genomes.

Positions of PII protein homologs were identified in the genome sequences through tblastn searches, using the GlnB protein of *A. brasilense* as query (ACF77123.1). Only hits presenting e-values equal to or lower than 1e-5 were considered. A sequence window containing the PII homolog sequence with its respective 10 kb upstream and 10 kb downstream sequences was defined as a “PII island.” Genome assemblies not presenting this sequence interval (contigs too short to contain 20 kb of adjacent sequences) were discarded. All protein sequences encoded in the “PII islands” were recovered, and they composed a database named as “PII neighbors DB.”

Homologs of the glutamine-dependent NadE proteins of *A. brasilense* (UniProt G8AIW8) and (UniProt G8ASI0) were searched for in the “PII neighbors DB” with blastp, using 1e-5 as e-value cutoff. All NadE-like protein sequences were recovered using fasgrep from FAST tools (55) and dereplicated using rmdup from Seqkit package (54). The distance between the midpoint of each *nadE* homolog gene and its closest neighboring PII homolog (midpoint of the PII island) was computed. Only *nadE* homologous sequences with the midpoint to PII homolog lower than 2000 bp were kept. Duplicated NCBI protein entries were removed. The remaining sequences were retrieved from NCBI and subjected to clustering analysis based on all against all BLAST+ similarities to detect orthologous groups using CLANS (33). The taxonomic affiliation of each NadE homolog was recorded using the data available in the NCBI2lin repository (https://github.com/zyxue/ncbitax2lin),^5^ which contains pre-converted lineages from the NCBI taxonomy database.

## Data availability statement

Additional data are available upon request: Prof. Luciano F. Huergo, Setor Litoral, UFPR, luciano.huergo@gmail.com.

## Author contributions

A. R. S. S., E. C. M. G., E. M. S., L. F. H., and K. F. conceptualization; A. R. S. S., L. M. P. P., L. F. H., and K. F. data curation; A. R. S. S., E. C. M. G., F. H. S., G. A. d. S., L. F. H., and K. F. formal analysis; A. R. S. S., E. C. M. G., E. P., F. H. S., G. A. d. S., L. F. H., and K. F. investigation; A. R. S. S., G. A. d. S., L. F. H., and K. F. methodology; E. C. M. G., M. B. R. S., L. S. C., L. M. P. P., L. F. H., and K. F. supervision; F. O. P., M. B. R. S., L. S. C., E. M. S., L. F. H., and K. F. funding acquisition; F. O. P., E. M. S., L. F. H., and K. F. project administration; F. H. S. and G. A. d. S. software; F. H. S., G. A. d. S., L. F. H., and K. F. writing-review and editing; L. F. H. resources; L. F. H. writing-original draft.

## Supplementary Material

Supporting Information

## Footnotes

5 Please note that the JBC is not responsible for the long-term archiving and maintenance of this site or any other third party hosted site.

## Footnotes

4 The abbreviations used are:

2-OG: 2-oxoglutarate
Ni-NTA: nickel-nitrilotriacetic acid
TEV: tobacco etch virus.

## References

[1] Houtkooper R. H., Cantó C., Wanders R. J., and Auwerx J. (2010) The secret life of NAD+: An old metabolite controlling new metabolic signaling pathways. Endocr. Rev. 31, 194–223 10.1210/er.2009-0026 20007326 PMC2852209

[2] Sorci L., Ruggieri S., and Raffaelli N. (2014) NAD homeostasis in the bacterial response to DNA/RNA damage. DNA Repair (Amst.) 23, 17–26 10.1016/j.dnarep.2014.07.014 25127744

[3] Jäschke A., Höfer K., Nübel G., and Frindert J. (2016) Cap-like structures in bacterial RNA and epitranscriptomic modification. Curr. Opin. Microbiol. 30, 44–49 10.1016/j.mib.2015.12.009 26779928

[4] Kurnasov O., Goral V., Colabroy K., Gerdes S., Anantha S., Osterman A., and Begley T. P. (2003) NAD biosynthesis: Identification of the tryptophan to quinolinate pathway in bacteria. Chem. Biol. 10, 1195–1204 10.1016/j.chembiol.2003.11.011 14700627

[5] De Ingeniis J., Kazanov M. D., Shatalin K., Gelfand M. S., Osterman A. L., and Sorci L. (2012) Glutamine versus ammonia utilization in the NAD synthetase family. PLoS One 7, e39115 10.1371/journal.pone.0039115 22720044 PMC3376133

[6] Laronde-Leblanc N., Resto M., and Gerratana B. (2009) Regulation of active site coupling in glutamine-dependent NAD + synthetase. Nat. Struct. Mol. Biol. 16, 421–429 10.1038/nsmb.1567 19270703

[7] Santos A. R. S., Gerhardt E. C. M., Moure V. R., Pedrosa F. O., Souza E. M., Diamanti R., Högbom M., and Huergo L. F. (2018) Kinetics and structural features of dimeric glutamine-dependent bacterial NAD synthetases suggest evolutionary adaptation to available metabolites. J. Biol. Chem. 293, 7397–7407 10.1074/jbc.RA118.002241 29581233 PMC5950007

[8] Grose J. H., Bergthorsson U., and Roth J. R. (2005) Regulation of NAD synthesis by the trifunctional NadR protein of *Salmonella enterica*. J. Bacteriol. 187, 2774–2782 10.1128/JB.187.8.2774-2782.2005 15805524 PMC1070365

[9] Rodionov D. A., De Ingeniis J., Mancini C., Cimadamore F., Zhang H., Osterman A. L., and Raffaelli N. (2008) Transcriptional regulation of NAD metabolism in bacteria: NrtR family of Nudix-related regulators. Nucleic Acids Res. 36, 2047–2059 10.1093/nar/gkn047 18276643 PMC2330246

[10] Dandekar T., Snel B., Huynen M., and Bork P. (1998) Conservation of gene order: A fingerprint of proteins that physically interact. Trends Biochem. Sci. 23, 324–328 10.1016/S0968-0004(98)01274-2 9787636

[11] Sant'Anna F. H., Trentini D. B., de Souto Weber S., Cecagno R., da Silva S. C., and Schrank I. S. (2009) The PII superfamily revised: A novel group and evolutionary insights. J. Mol. Evol. 68, 322–336 10.1007/s00239-009-9209-6 19296042

[12] Huergo L. F., Chandra G., and Merrick M. (2013) PII signal transduction proteins: Nitrogen regulation and beyond. FEMS Microbiol. Rev. 37, 251–283 10.1111/j.1574-6976.2012.00351.x 22861350

[13] Arcondéguy T., Jack R., and Merrick M. (2001) P(II) signal transduction proteins, pivotal players in microbial nitrogen control. Microbiol. Mol. Biol. Rev. 65, 80–105 10.1128/MMBR.65.1.80-105.2001 11238986 PMC99019

[14] Forchhammer K., and Lüddecke J. (2016) Sensory properties of the PII signalling protein family. FEBS J. 283, 425–437 10.1111/febs.13584 26527104

[15] Huergo L. F., and Dixon R. (2015) The emergence of 2-oxoglutarate as a master regulator metabolite. Microbiol. Mol. Biol. Rev. 79, 419–435 10.1128/MMBR.00038-15 26424716 PMC4651028

[16] Jiang P., and Ninfa A. J. (2007) *Escherichia coli* PII signal transduction protein controlling nitrogen assimilation acts as a sensor of adenylate energy charge in vitro. Biochemistry 46, 12979–12996 10.1021/bi701062t 17939683

[17] Truan D., Huergo L. F., Chubatsu L. S., Merrick M., Li X.-D., and Winkler F. K. (2010) A new PII protein structure identifies the 2-oxoglutarate binding site. J. Mol. Biol. 400, 531–539 10.1016/j.jmb.2010.05.036 20493877

[18] Fokina O., Chellamuthu V.-R., Forchhammer K., and Zeth K. (2010) Mechanism of 2-oxoglutarate signaling by the Synechococcus elongatus PII signal transduction protein. Proc. Natl. Acad. Sci. U.S.A. 107, 19760–19765 10.1073/pnas.1007653107 21041661 PMC2993416

[19] Oliveira M. A. S., Gerhardt E. C. M., Huergo L. F., Souza E. M., Pedrosa F. O., and Chubatsu L. S. (2015) 2-Oxoglutarate levels control adenosine nucleotide binding by Herbaspirillum seropedicae PII proteins. FEBS J. 282, 4797–4809 10.1111/febs.13542 26433003

[20] Da Rocha R. A., Weschenfelder T. A., De Castilhos F., De Souza E. M., Huergo L. F., and Mitchell D. A. (2013) Mathematical model of the binding of allosteric effectors to the Escherichia coli PII signal transduction protein GlnB. Biochemistry 52, 2683–2693 10.1021/bi301659r 23517273

[21] Merrick M. (2015) Post-translational modification of PII signal transduction proteins. Front. Microbiol. 5, 763 10.3389/fmicb.2014.00763 25610437 PMC4285133

[22] Chellamuthu V. R., Ermilova E., Lapina T., Lüddecke J., Minaeva E., Herrmann C., Hartmann M. D., and Forchhammer K. (2014) A widespread glutamine-sensing mechanism in the plant kingdom. Cell 159, 1188–1199 10.1016/j.cell.2014.10.015 25416954

[23] Bennett B. D., Kimball E. H., Gao M., Osterhout R., Van Dien S. J., and Rabinowitz J. D. (2009) Absolute metabolite concentrations and implied enzyme active site occupancy in *Escherichia coli*. Nat. Chem. Biol. 5, 593–599 10.1038/nchembio.186 19561621 PMC2754216

[24] Huergo L. F., Souza E. M., Araujo M. S., Pedrosa F. O., Chubatsu L. S., Steffens M. B. R., and Merrick M. (2006) ADP-ribosylation of dinitrogenase reductase in *Azospirillum brasilense* is regulated by AmtB-dependent membrane sequestration of DraG. Mol. Microbiol. 59, 326–337 10.1111/j.1365-2958.2005.04944.x 16359338

[25] de Zamaroczy M. (1998) Structural homologues PII and PZ of *Azospirillum brasilense* provide intracellular signalling for selective regulation of various nitrogen-dependent functions. Mol. Microbiol. 29, 449–463 10.1046/j.1365-2958.1998.00938.x 9720864

[26] Wu C. H. (2004) PIRSF: family classification system at the Protein Information Resource. Nucleic Acids Res. 32, D112–D114 10.1093/nar/gkh097 14681371 PMC308831

[27] Rodionova I. A., Schuster B. M., Guinn K. M., Sorci L., Scott D. A., Li X., Kheterpal I., Shoen C., Cynamon M., Locher C., Rubin E. J., and Osterman A. L. (2014) Metabolic and bactericidal effects of targeted suppression of NadD and NadE enzymes in mycobacteria. MBio 5, e000747–13 10.1128/mBio.00747-13 24549842 PMC3944813

[28] van Heeswijk W. C., Westerhoff H. V., and Boogerd F. C. (2013) Nitrogen assimilation in *Escherichia coli*: Putting molecular data into a systems perspective. Microbiol. Mol. Biol. Rev. 77, 628–695 10.1128/MMBR.00025-13 24296575 PMC3973380

[29] Yuan J., Doucette C. D., Fowler W. U., Feng X. J., Piazza M., Rabitz H. A., Wingreen N. S., and Rabinowitz J. D. (2009) Metabolomics-driven quantitative analysis of ammonia assimilation in *E. coli*. Mol. Syst. Biol. 5, 302 10.1038/msb.2009.60 19690571 PMC2736657

[30] Huergo L. F., Pedrosa F. O., Muller-Santos M., Chubatsu L. S., Monteiro R. A., Merrick M., and Souza E. M. (2012) P II signal transduction proteins: Pivotal players in post-translational control of nitrogenase activity. Microbiology 158, 176–190 10.1099/mic.0.049783-0 22210804

[31] Moure V. R., Danyal K., Yang Z.-Y., Wendroth S., Müller-Santos, Pedrosa M. F. O., Scarduelli M., Gerhardt E. C. M., Huergo L. F., Souza E. M., and Seefeldta L. C. (2013) The nitrogenase regulatory enzyme dinitrogenase reductase adpribosyltransferase (DraT) is activated by direct interaction with the signal transduction protein glnb. J. Bacteriol. 195, 279–286 10.1128/JB.01517-12 23144248 PMC3553828

[32] Moure V. R., Costa F. F., Cruz L. M., Pedrosa F. O., Souza E. M., Li X. D., Winkler F., and Huergo L. F. (2014) Regulation of nitrogenase by reversible mono-ADP-ribosylation. Curr. Top. Microbiol. Immunol. 384, 89–106 10.1007/82_2014_380 24934999

[33] Frickey T., and Lupas A. (2004) CLANS: A Java application for visualizing protein families based on pairwise similarity. Bioinformatics 20, 3702–3704 10.1093/bioinformatics/bth444 15284097

[34] Zeth K., Fokina O., and Forchhammer K. (2014) Structural basis and target-specific modulation of ADP sensing by the *Synechococcus elongatus* PII signaling protein. J. Biol. Chem. 289, 8960–8972 10.1074/jbc.M113.536557 24519945 PMC3979405

[35] Coutts G., Thomas G., Blakey D., and Merrick M. (2002) Membrane sequestration of the signal transduction protein GlnK by the ammonium transporter AmtB. EMBO J. 21, 536–545 10.1093/emboj/21.4.536 11847102 PMC125854

[36] Gerhardt E. C. M., Rodrigues T. E., Müller-Santos M., Pedrosa F. O., Souza E. M., Forchhammer K., and Huergo L. F. (2015) The bacterial signal transduction protein GlnB regulates the committed step in fatty acid biosynthesis by acting as a dissociable regulatory subunit of acetyl-CoA carboxylase. Mol. Microbiol. 95, 1025–1035 10.1111/mmi.12912 25557370

[37] Rodrigues T. E., Sassaki G. L., Valdameri G., Pedrosa F. O., Souza E. M., and Huergo L. F. (2019) Fatty acid biosynthesis is enhanced in *Escherichia coli* strains with deletion in genes encoding the PII signaling proteins. Arch. Microbiol. 201, 209–214 10.1007/s00203-018-1603-2 30506165

[38] Zhang Z., Milias-Argeitis A., and Heinemann M. (2018) Dynamic single-cell NAD(P)H measurement reveals oscillatory metabolism throughout the *E. coli* cell division cycle. Sci. Rep. 8, 2162 10.1038/s41598-018-20550-7 29391569 PMC5795003

[39] Strømland Ø., Ziegler M., Heiland I., VanLinden M. R., Nikiforov A. A., and Niere M. (2019) Keeping the balance in NAD metabolism. Biochem. Soc. Trans. 47, 119–130 10.1042/bst20180417 30626706

[40] Cahová H., Winz M. L., Höfer K., Nübel G., and Jäschke A. (2015) NAD captureSeq indicates NAD as a bacterial cap for a subset of regulatory RNAs. Nature 519, 374–377 10.1038/nature14020 25533955

[41] Burckhardt R. M., Buckner B. A., and Escalante-Semerena J. C. (2019) *Staphylococcus aureus* modulates the activity of acetyl-coenzyme A synthetase (Acs) by sirtuin-dependent reversible lysine acetylation. Mol. Microbiol. 112, 588–604 10.1111/mmi.14276 31099918 PMC6703943

[42] Lin H. (2007) Nicotinamide adenine dinucleotide: Beyond a redox coenzyme. Org. Biomol. Chem. 5, 2541–2554 10.1039/b706887e 18019526

[43] Gravina F., Sanchuki H. S., Rodrigues T. E., Gerhardt E. C. M., Pedrosa F. O., Souza E. M., Valdameri G., de Souza G. A., and Huergo L. F. (2018) Proteome analysis of an *Escherichia coli* ptsN-null strain under different nitrogen regimes. J. Proteomics 174, 28–35 10.1016/j.jprot.2017.12.006 29274402

[44] Gerdes S. Y., Kurnasov O. V., Shatalin K., Polanuyer B., Sloutsky R., Vonstein V., Overbeek R., and Osterman A. L. (2006) Comparative genomics of NAD biosynthesis in cyanobacteria. J. Bacteriol. 188, 3012–3023 10.1128/JB.188.8.3012-3023.2006 16585762 PMC1446974

[45] Rajendran C., Gerhardt E. C. M., Bjelic S., Gasperina A., and Scarduelli M. (2011) Crystal structure of the GlnZ-DraG complex reveals a different form of P II -target interaction. Proc. Natl. Acad. Sci. 108, 18972–18976 10.1073/pnas.1108038108 22074780 PMC3223478

[46] van den Berg S., Löfdahl P. Å., Härd T., Berglund H. (2006) Improved solubility of TEV protease by directed evolution. J. Biotechnol. 121, 291–298 10.1016/j.jbiotec.2005.08.006 16150509

[47] Araújo L. M., Huergo L. F., Invitti A. L., Gimenes C. I., Bonatto A. C., Monteiro R. A., Souza E. M., Pedrosa F. O., and Chubatsu L. S. (2008) Different responses of the GlnB and GlnZ proteins upon *in vitro* uridylylation by the *Azospirillum brasilense* GlnD protein. Braz. J. Med. Biol. Res. 41, 289–294 10.1590/S0100-879X2008000400006 18392451

[48] Rippka R., Deruelles J., Waterbury J. B., Michael Herdman M., and Stanier R. Y. (1979) Generic assignments, strain histories and properties of pure cultures of cyanobacteria. J. Gen. Microbiol. 111, 1–61 10.1099/00221287-111-1-1

[49] Pedrosa F. O., and Yates M. G. (1984) Regulation of nitrogen fixation (*nif*) genes of *Azospirillum brasilense* by *nifA* and *ntr* (*gln*) type gene products. FEMS Microbiol. Lett. 23, 95–101 10.1111/j.1574-6968.1984.tb01042.x

[50] Huergo L. F., Chubatsu L. S., Souza E. M., Pedrosa F. O., Steffens M. B. R., and Merrick M. (2006) Interactions between PII proteins and the nitrogenase regulatory enzymes DraT and DraG in *Azospirillum brasilense*. FEBS Lett. 580, 5232–5236 10.1016/j.febslet.2006.08.054 16963029

[51] Rocco C. J., Dennison K. L., Klenchin V. A., Rayment I., and Escalante-Semerena J. C. (2008) Construction and use of new cloning vectors for the rapid isolation of recombinant proteins from *Escherichia coli*. Plasmid 59, 231–237 10.1016/j.plasmid.2008.01.001 18295882 PMC2386272

[52] Moure V. R., Razzera G., Araújo L. M., Oliveira M. A. S., Gerhardt E. C. M., Müller-Santos M., Almeida F., Pedrosa F. O., Valente A. P., Souza E. M., and Huergo L. F. (2012) Heat stability of Proteobacterial PII protein facilitate purification using a single chromatography step. Protein Expr. Purif. 81, 83–88 10.1016/j.pep.2011.09.008 21963770

[53] Araujo M. S., Baura V. A., Souza E. M., Benelli E. M., Rigo L. U., Steffens M. B. R., Pedrosa F. O., and Chubatsu L. S. (2004) *In vitro* uridylylation of the *Azospirillum brasilense* N-signal transducing GlnZ protein. Protein Expr. Purif. 33, 19–24 10.1016/j.pep.2003.08.024 14680957

[54] Shen W., Le S., Li Y., and Hu F. (2016) SeqKit: A cross-platform and ultrafast toolkit for FASTA/Q file manipulation. PLoS One 11, e0163962 10.1371/journal.pone.0163962 27706213 PMC5051824

[55] Lawrence T. J., Kauffman K. T., Amrine K. C. H., Carper D. L., Lee R. S., Becich P. J., Canales C. J., and Ardell D. H. (2015) FAST: FAST Analysis of Sequences Toolbox. Front. Genet. 6, 172 10.3389/fgene.2015.00172 26042145 PMC4437040

