# Supplementary material for: NAD+ biosynthesis in bacteria is controlled by global carbon/nitrogen levels via PII signaling
Source: J Biol Chem. 2020 Mar 16;295(18):6165–76. doi: 10.1074/jbc.RA120.012793 (PMC7196632; doi:10.1074/jbc.RA120.012793)
Supplement: Supporting Information [file supp_295_18_6165__index.html]

NAD+ biosynthesis in bacteria is controlled by global carbon/nitrogen levels via PII signaling — PII regulates NAD+ biosynthesis bacteria — NAD+ biosynthesis in bacteria is controlled by global carbon/nitrogen levels via PII signaling — PII regulates NAD+ biosynthesis in bacteria — Supporting Information 

# NAD+ biosynthesis in bacteria is controlled by global carbon/nitrogen levels via PII signaling

## Supporting Information

- Table S1 - Table S1 - Ocurrence of PII-nadE genetic islands in sequenced prokaryote genomes.
- Supporting Information (to be published online) - Supporting Tables and Figures
