# Supplementary material for: NAD+ biosynthesis in bacteria is controlled by global carbon/nitrogen levels via PII signaling
Source: J Biol Chem. 2020 Mar 16;295(18):6165–76. doi: 10.1074/jbc.RA120.012793 (PMC7196632; doi:10.1074/jbc.RA120.012793)
Supplement: Supporting Information [file supp_RA120.012793_158430_2_supp_492850_q7wd2b.pdf]

Supporting Information

NAD$^+$ biosynthesis in Bacteria is subject to global Carbon/Nitrogen control via PII signaling

Adrian Richard Schenberger Santos[1,3], Edileusa Cristina Marques Gerhardt[1,] Erick Parize[1], Fabio Oliveira Pedrosa[1], Maria Berenice Reynaud Steffens[1], Leda Satie Chubatsu[1], Emanuel Maltempi Souza[1], Luciane Maria Pereira Passaglia[4], Fernando Hayashi Sant´Anna[4], Gustavo Antônio de Souza[5], Luciano Fernandes Huergo[1,2,3]* and Karl Forchhammer[3]*

From the: [1]Departamento de Bioquímica e Biologia Molecular, UFPR Curitiba, PR, Brazil; [2]Setor Litoral, UFPR Matinhos, PR, Brazil; [3]Interfakultäres Institut für Mikrobiologie und Infektionsmedizin der Eberhard-Karls Universität Tübingen, Auf der Morgenstelle 28, Tübingen 72076, Germany; [4]Departamento de Genética, Instituto de Biociências, Universidade Federal do Rio Grande do Sul, Porto Alegre, Brazil; [5]Departamento de Bioquímica, Universidade Federal do Rio Grande do Norte, Natal, RN, Brazil.

Table S2 - Bacterial strains and plasmids

Strain/plasmid	Genotype/phenotype	Source/reference
***Synechocystis* sp.**		
PCC 6803	Wild-type	(49)
A. brasilense		
FP2	Wild-type	(50)
LFH3	Nal^r, Δ*glnB* Nif$^-$	(51)
7611	Nif$^+$ *glnZ* ::Ω, Sp^r Sm^r	(25)
2812	*glnB*::kan/ *glnZ*:: Ω , Sm^r Km^r	(25)
E . coli		
DH10B	Sm^r ; F$'$ [*pro*AB$^+$ *lac*ZM15]	Invitrogen
Rosetta (DE) pLysS	F- ompT hsdSB(rB- mB-) gal dcm (DE3) pLysSRARE	Novagen
Lemo21 (DE3)	Expresses T7 RNA polymerase	New England Labs Inc.
**Plasmid**	**Characteristics**	**Reference**
pET28a	Km^r Expression vector	Agilent
pET29a	Km^r Expression vector	Agilent
pTEV5	Amp^r Expression vector, His tag followed by TEV cleavage site	(52)
pASnade2	Km^r. Express *H. seropedicae* NadE2 with a N-terminal 6x His tag in pET28a	(7)

pLHnade2	Km^r. Express native *A. brasilense* NadE2 in pET29a	(7)
pASnadESc	Amp^r. Express *Synechocystis* sp. strain PCC 6803 NadE with a N-terminal 6x His tag in pTEV5	This work
pMSA4Δ42-54	Km^r. Express native *A. brasilense* GlnZΔ42-54 in pET29a	(45)
pGlnZΔTloop	Km^r. Express *A. brasilense* GlnZΔ42-54 with a N-terminal 6x His tag in pET28a	This work
pMSA3	Km^r. Express *A.brasilense* GlnZ with a N-terminal 6x His tag in pET28a	(53)
pMSA4	Km^r. Express native *A. brasilense* GlnZ in pET29a	(54)
pLH25	Km^r. Express *A.brasilense* GlnB with a N-terminal 6x His tag in pET28a	(55)
pASGlnDAb	Amp^r. Express *A. brasilense* GlnD with a N-terminal 6x His tag in pTEV5	This work

Supporting Figures

A

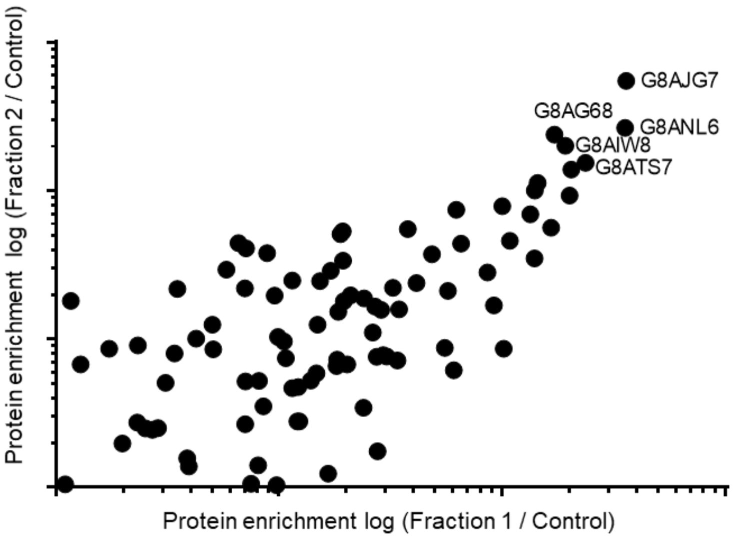

B

		Enrichment (log10)	
**Uniprot**	**Description**	**Fraction1/Control**	**Fraction2/Control**
**G8AJG7**	Orotate phosphoribosyltransferase	3,25	3,54
**G8ANL6**	Uncharacterized protein	3,24	3,06
**G8AG68**	Putative Phosphate dikinase regulatory protein	2,80	2,99
**G8AIW8**	Glutamine-dependent NAD(+) synthetase (NadE)	2,86	2,89
**G8ATS7**	Uncharacterized protein	2,99	2,74

Figure S1. Putative binding partners of the *A. brasilense* PII protein GlnZ. A) A 1 ml Ni-NTA column charged with His-tagged GlnZ protein was loaded with *A. brasilense* protein extracts in the presence of MgATP. After extensive washes, proteins were eluted with buffer containing MgATP and 1.5 mM of 2-OG and separated in 1.5 ml fractions (fractions 1 and 2). The amount of the proteins in each fraction was compared to the amount of proteins eluted from a control column, under the same conditions, by quantitative label free LC/MS/MS in technical triplicates. The graphic shows the log protein enrichment of the signal of each protein eluted the His-GlnZ column / control column in their respective 1.5 mL fractions. **B)** Details of the top 5 enriched proteins that were eluted from the His-GlnZ affinity column.

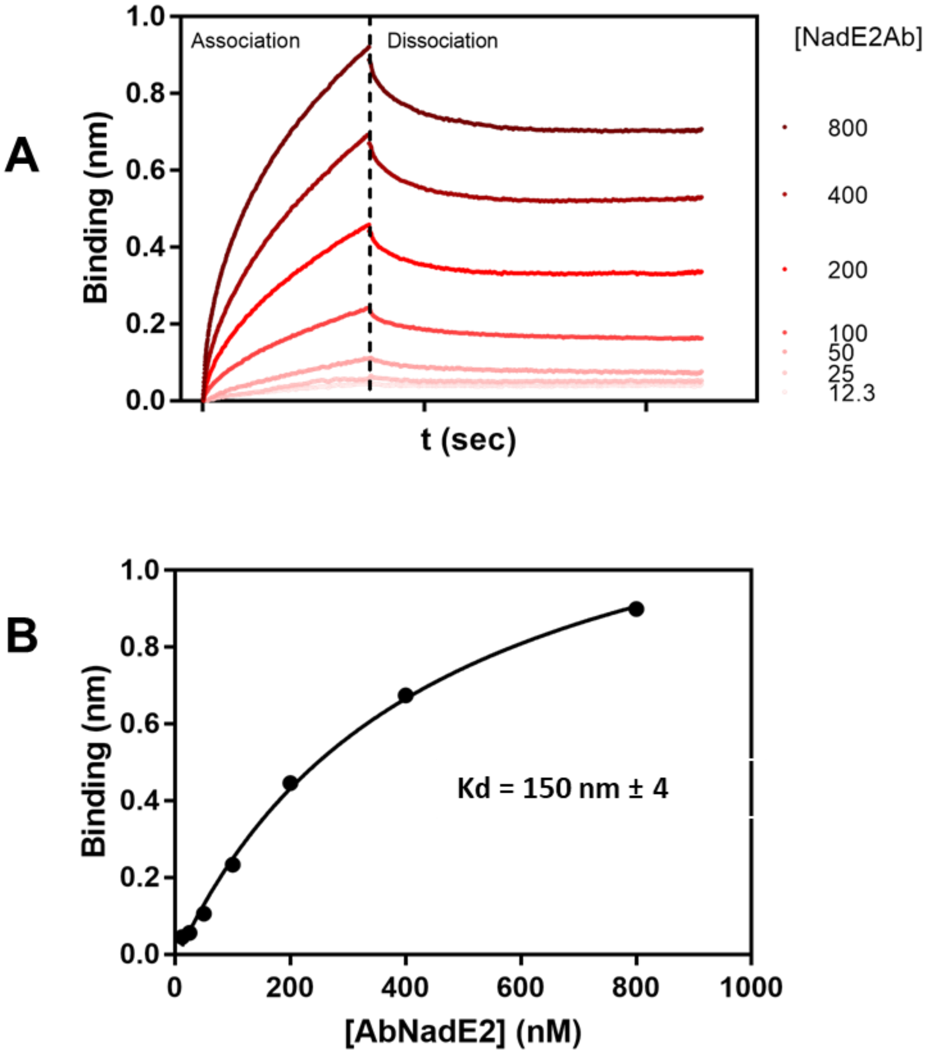

Figure S2. Determination of the dissociation constant of the His-GlnZ and AbNadE2 complex in the presence of ADP by Bio-layer Interferometry. A) The His-GlnZ was immobilized in the Ni-NTA sensor tip in a concentration of 2 µg/mL until saturation. The tip with His-GlnZ was then challenged in solution containing the indicated AbNadE2 concentrations in the presence of 1mM ADP. **B)** Plot reporting the Δλ spectral shift in nm vs AbNadE2 concentration. The binding was measured in an Octet K2 (Fortébio) and the Kd determined using the manufacturer´s software.

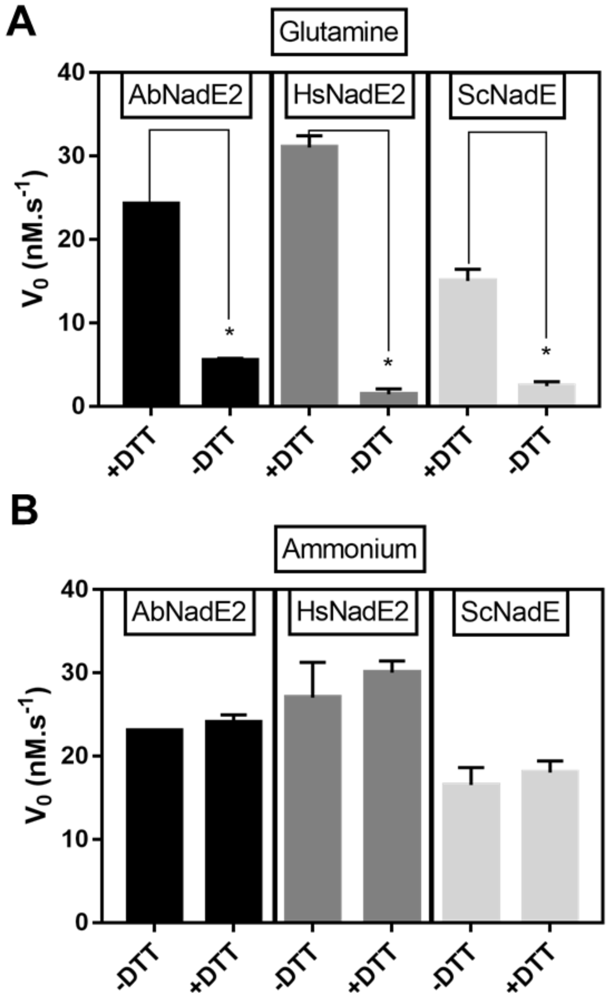

Figure S3. The NadE^{Gln} type 2 are activated in reductive environment when using L-glutamine as N-donor. NadE^{Gln} type 2 activity was continuously measured by determining NADH formation in assays coupled with alcohol dehydrogenase in the presence or absence of 10 mM Dithiothreitol (DTT) using 2 mM of L-glutamine as a N-donor (**A**) or 10 mM of ammonium as a N-donor **(B**)**.** Proteins from *A. brasilense* (AbNadE2), *Herbaspirillum seropedicae* (HsNadE2) and *Synechocystis* sp. (ScNadE2) were assayed.

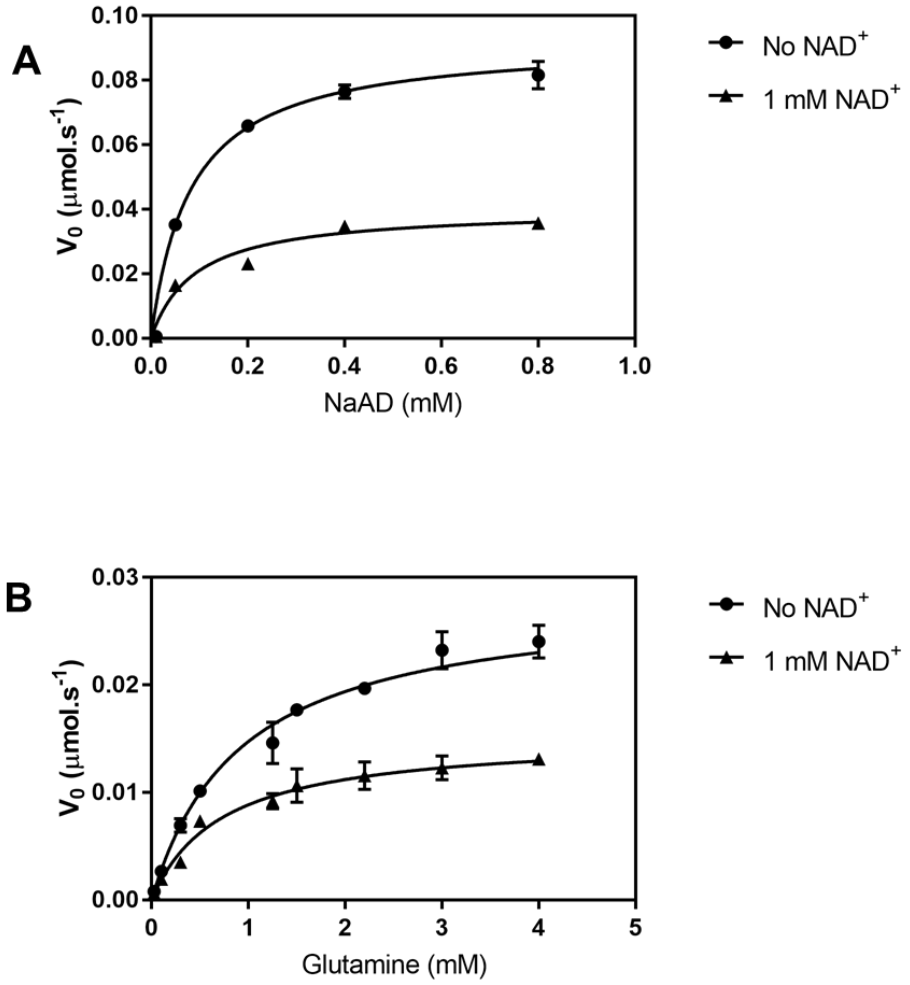

Figure S4. Effect of NAD^+ in the kinetic parameters of AbNadE2 reaction. A) Determination of initial velocities accordingly to varying NaAD concentrations in the presence and absence of 1mM NAD^+. A NaAD K_M of 0.08 mM and Vmax of 0.09 µmol.s^{-1} were obtained in the absence of NAD^+. In the presence of NAD^+ the values were K_M 0.09 mM and Vmax 0.04 µmol.s^{-1} . **B)** Determination of initial velocities accordingly to varying L-glutamine concentrations in the presence and absence of 1 mM NAD^+. A L-glutamine K_M of 0.72 mM and Vmax of 0.15 µmol.s^{-1} were obtained in the absence of NAD^+. In the presence of NAD^+ the values were K_M 0.92 mM and Vmax 0.028 µmol.s^{-1} .

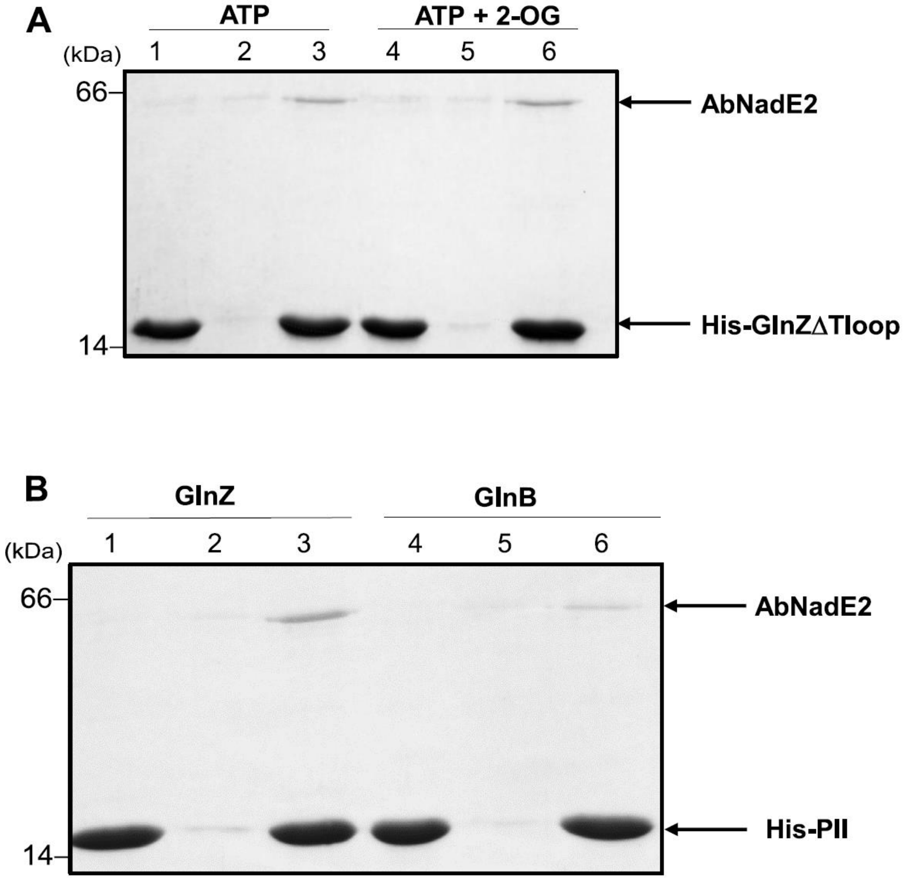

Figure S5. *In vitro* formation of the NadE2-GlnZ complex does not require the GlnZ T loop and AbNadE2 preferentially interacts with the PII protein GlnZ. **A)** Complex formation was assessed by pull-down using Ni^{2+} beads. Reactions were performed in the presence of the ATP and 2-OG at 1mM, as indicated. Binding reactions were conducted in 400 µl of buffer (50 mM Tris HCl; 100 mM NaCl; glycerol 10%; 20 mM Imidazole; 0,05% Tween 20; 5 mM $MgCl_2$). The fractions eluted from the Ni^{2+} beads were analyzed by SDS-PAGE and the gel was stained with Coomassie Blue. Lanes 1 and 4, HisGlnZ only. Lanes 2 and 5, AbNadE2 only. Lanes 3 and 6, mixture of HisGlnZ and AbNadE2. **B)** Complex formation was assessed by pull-down using Ni^{2+} beads. Reactions were performed under fixed concentration ATP 1 mM. Binding reactions were conducted in 400 µl of buffer (50 mM Tris HCl; 100 mM NaCl; glycerol 10%; 20 mM Imidazole; 0,05% Tween 20; 5 mM $MgCl_2$). The fractions eluted from the Ni^{2+} beads were analyzed by SDS-PAGE 15% and the gel was stained with Coomassie Blue. Lanes 1 and 4, His-PII only (GlnB or GlnZ as indicated). Lanes 2 and 5, AnNadE2 only. Lanes 3 and 6, mixture of His-PII and AbNadE2.

References of Supporting Information

1. Houtkooper, R. H., Cantó, C., Wanders, R. J., and Auwerx, J. (2010) The secret life of NAD+: An old metabolite controlling new metabolic signaling pathways. *Endocr. Rev.* **31**, 194–223

2. Sorci, L., Ruggieri, S., and Raffaelli, N. (2014) NAD homeostasis in the bacterial response to DNA/RNA damage. *DNA Repair (Amst).* 10.1016/j.dnarep.2014.07.014

3. Jäschke, A., Höfer, K., Nübel, G., and Frindert, J. (2016) Cap-like structures in bacterial RNA and epitranscriptomic modification. *Curr. Opin. Microbiol.* **30**, 44–49

4. Kurnasov, O., Goral, V., Colabroy, K., Gerdes, S., Anantha, S., Osterman, A., and Begley, T. P. (2003) NAD Biosynthesis: Identification of the Tryptophan to Quinolinate Pathway in Bacteria. *Chem. Biol.* 10.1016/j.chembiol.2003.11.011

5. De Ingeniis, J., Kazanov, M. D., Shatalin, K., Gelfand, M. S., Osterman, A. L., and Sorci, L. (2012) Glutamine versus ammonia utilization in the NAD synthetase family. *PLoS One*. **7**, e39115

6. Laronde-Leblanc, N., Resto, M., and Gerratana, B. (2009) Regulation of active site coupling in glutamine-dependent NAD + synthetase. *Nat. Struct. Mol. Biol.* 10.1038/nsmb.1567

7. Santos, A. R. S., Gerhardt, E. C. M., Moure, V. R., Pedrosa, F. O., Souza, E. M., Diamanti, R., Högbom, M., and Huergo, L. F. (2018) Kinetics and structural features of dimeric glutamine-dependent bacterial NAD synthetases suggest evolutionary adaptation to available metabolites. *J. Biol. Chem.* 10.1074/jbc.RA118.002241

8. Grose, J. H., Bergthorsson, U., and Roth, J. R. (2005) Regulation of NAD synthesis by the trifunctional NadR protein of Salmonella enterica. *J. Bacteriol.* **187**, 2774–2782

9. Rodionov, D. a, De Ingeniis, J., Mancini, C., Cimadamore, F., Zhang, H., Osterman, A. L., and Raffaelli, N. (2008) Transcriptional regulation of NAD metabolism in bacteria: NrtR family of Nudix-related regulators. *Nucleic Acids Res.* **36**, 2047–59

10. Dandekar, T., Snel, B., Huynen, M., and Bork, P. (1998) Conservation of gene order: A fingerprint of proteins that physically interact. *Trends Biochem. Sci.* 10.1016/S0968-0004(98)01274-2

11. Sant'Anna, F. H., Trentini, D. B., de Souto Weber, S., Cecagno, R., da Silva, S. C., and Schrank, I. S. (2009) The PII superfamily revised: a novel group and evolutionary insights. *J. Mol. Evol.* **68**, 322–36

12. Huergo, L. F., Chandra, G., and Merrick, M. (2013) PII signal transduction proteins: Nitrogen regulation and beyond. *FEMS Microbiol. Rev.* 10.1111/j.1574-6976.2012.00351.x

13. Arcondéguy, T., Jack, R., and Merrick, M. (2001) P(II) signal transduction proteins, pivotal players in microbial nitrogen control. *Microbiol. Mol. Biol. Rev.* **65**, 80–105

14. Forchhammer, K., and Lüddecke, J. (2016) Sensory properties of the PII signalling protein family. *FEBS J.* **283**, 425–437

15. Huergo, L. F., and Dixon, R. (2015) The Emergence of 2-Oxoglutarate as a Master Regulator Metabolite. *Microbiol. Mol. Biol. Rev.* **79**, 419–435

16. Jiang, P., and Ninfa, A. J. (2007) Escherichia coli PII signal transduction protein controlling nitrogen assimilation acts as a sensor of adenylate energy charge in vitro. *Biochemistry.* 10.1021/bi701062t

17. Truan, D., Huergo, L. F., Chubatsu, L. S., Merrick, M., Li, X.-D., and Winkler, F. K. (2010) A New PII Protein Structure Identifies the 2-Oxoglutarate Binding Site. *J. Mol. Biol.* 10.1016/j.jmb.2010.05.036

18. Fokina, O., Chellamuthu, V.-R., Forchhammer, K., and Zeth, K. (2010) Mechanism of 2-oxoglutarate signaling by the Synechococcus elongatus PII signal transduction protein. *Proc. Natl. Acad. Sci.*

10.1073/pnas.1007653107

19. Oliveira, M. A. S., Gerhardt, E. C. M., Huergo, L. F., Souza, E. M., Pedrosa, F. O., and Chubatsu, L. S. (2015) 2-Oxoglutarate levels control adenosine nucleotide binding by Herbaspirillum seropedicae PII proteins. *FEBS J.* 10.1111/febs.13542

20. Da Rocha, R. A., Weschenfelder, T. A., De Castilhos, F., De Souza, E. M., Huergo, L. F., and Mitchell, D. A. (2013) Mathematical model of the binding of allosteric effectors to the escherichia coli PII signal transduction protein GlnB. *Biochemistry*. 10.1021/bi301659r

21. Merrick, M. (2015) Post-translational modification of PII signal transduction proteins. *Front. Microbiol.* **5**, 763

22. Chellamuthu, V. R., Ermilova, E., Lapina, T., L??ddecke, J., Minaeva, E., Herrmann, C., Hartmann, M. D., and Forchhammer, K. (2014) A widespread glutamine-sensing mechanism in the plant kingdom. *Cell*. **159**, 1188–1199

23. Bennett, B. D., Kimball, E. H., Gao, M., Osterhout, R., Van Dien, S. J., and Rabinowitz, J. D. (2009) Absolute metabolite concentrations and implied enzyme active site occupancy in Escherichia coli. *Nat. Chem. Biol.* **5**, 593–599

24. Huergo, L. F., Souza, E. M., Araujo, M. S., Pedrosa, F. O., Chubatsu, L. S., Steffens, M. B. R., and Merrick, M. (2006) ADP-ribosylation of dinitrogenase reductase in Azospirillum brasilense is regulated by AmtB-dependent membrane sequestration of DraG. *Mol. Microbiol.* 10.1111/j.1365-2958.2005.04944.x

25. De Zamaroczy, M. (1998) Structural homologues PII and PZ of Azospirillum brasilense provide intracellular signalling for selective regulation of various nitrogen-dependent functions. *Mol. Microbiol.* **29**, 449–463

26. Wu, C. H. (2004) PIRSF: family classification system at the Protein Information Resource. *Nucleic Acids Res.* 10.1093/nar/gkh097

27. Rodionova, I. A., Schuster, B. M., Guinn, K. M., Sorci, L., Scott, D. A., Li, X., Kheterpal, I., Shoen, C., Cynamon, M., Locher, C., Rubin, E. J., and Osterman, A. L. (2014) Metabolic and bactericidal effects of targeted suppression of NadD and NadE enzymes in mycobacteria. *MBio*. 10.1128/mBio.00747-13

28. van Heeswijk, W. C., Westerhoff, H. V, and Boogerd, F. C. (2013) Nitrogen assimilation in Escherichia coli: putting molecular data into a systems perspective. *Microbiol. Mol. Biol. Rev.* **77**, 628–95

29. Yuan, J., Doucette, C. D., Fowler, W. U., Feng, X. J., Piazza, M., Rabitz, H. A., Wingreen, N. S., and Rabinowitz, J. D. (2009) Metabolomics-driven quantitative analysis of ammonia assimilation in E. coli. *Mol. Syst. Biol.* 10.1038/msb.2009.60

30. Huergo, L. F., Pedrosa, F. O., Muller-Santos, M., Chubatsu, L. S., Monteiro, R. A., Merrick, M., and Souza, E. M. (2012) P II signal transduction proteins: Pivotal players in post-translational control of nitrogenase activity. *Microbiology*. 10.1099/mic.0.049783-0

31. Moure, V. R., Danyal, K., Yang, Z.-Y., Wendroth, S., Müller-santos, M., Pedrosa, F. O., Scarduelli, M., Gerhardt, E. C. M., Huergo, L. F., Souza, E. M., and Seefeldta, L. C. (2013) The nitrogenase regulatory enzyme dinitrogenase reductase adpribosyltransferase (DraT) is activated by direct interaction with the signal transduction protein glnb. *J. Bacteriol.* 10.1128/JB.01517-12

32. Moure, V. R., Costa, F. F., Cruz, L. M., Pedrosa, F. O., Souza, E. M., Li, X. D., Winkler, F., and Huergo, L. F. (2014) Regulation of nitrogenase by reversible mono-ADP-ribosylation. in *Current Topics in Microbiology and Immunology*, 10.1007/82_2014_380

33. Forchhammer, K., and Lüddecke, J. (2016) Sensory properties of the P_{II} signalling protein family. *FEBS J.* **283**, 425–437

34. Zeth, K., Fokinas, O., and Forchhammers, K. (2014) Structural basis and target-specific modulation

of ADP sensing by the Synechococcus elongatus PII signaling protein. *J. Biol. Chem.* **289**, 8960–8972

35. Coutts, G., Thomas, G., Blakey, D., and Merrick, M. (2002) Membrane sequestration of the signal transduction protein GlnK by the ammonium transporter AmtB. *EMBO J.* **21**, 536–45

36. Gerhardt, E. C. M., Rodrigues, T. E., Müller-Santos, M., Pedrosa, F. O., Souza, E. M., Forchhammer, K., and Huergo, L. F. (2015) The Bacterial signal transduction protein GlnB regulates the committed step in fatty acid biosynthesis by acting as a dissociable regulatory subunit of acetyl-CoA carboxylase. *Mol. Microbiol.* 10.1111/mmi.12912

37. Rodrigues, T. E., Sassaki, G. L., Valdameri, G., Pedrosa, F. O., Souza, E. M., and Huergo, L. F. (2019) Fatty acid biosynthesis is enhanced in Escherichia coli strains with deletion in genes encoding the PII signaling proteins. *Arch. Microbiol.* 10.1007/s00203-018-1603-2

38. Zhang, Z., Milias-Argeitis, A., and Heinemann, M. (2018) Dynamic single-cell NAD(P)H measurement reveals oscillatory metabolism throughout the E. coli cell division cycle. *Sci. Rep.* 10.1038/s41598-018-20550-7

39. Strømland, Ø., Ziegler, M., Heiland, I., VanLinden, M. R., Nikiforov, A. A., and Niere, M. (2019) Keeping the balance in NAD metabolism. *Biochem. Soc. Trans.* 10.1042/bst20180417

40. Cahová, H., Winz, M. L., Höfer, K., Nübel, G., and Jäschke, A. (2015) NAD captureSeq indicates NAD as a bacterial cap for a subset of regulatory RNAs. *Nature*. **519**, 374–377

41. Burckhardt, R. M., Buckner, B. A., and Escalante-Semerena, J. C. (2019) Staphylococcus aureus modulates the activity of acetyl-Coenzyme A synthetase (Acs) by sirtuin-dependent reversible lysine acetylation . *Mol. Microbiol.* 10.1111/mmi.14276

42. Lin, H. (2007) Nicotinamide adenine dinucleotide: Beyond a redox coenzyme. *Org. Biomol. Chem.* 10.1039/b706887e

43. Gravina, F., Sanchuki, H. S., Rodrigues, T. E., Gerhardt, E. C. M., Pedrosa, F. O., Souza, E. M., Valdameri, G., de Souza, G. A., and Huergo, L. F. (2018) Proteome analysis of an Escherichia coli ptsN-null strain under different nitrogen regimes. *J. Proteomics*. **174**, 28–35

44. Gerdes, S. Y., Kurnasov, O. V, Shatalin, K., Polanuyer, B., Sloutsky, R., Vonstein, V., Overbeek, R., and Osterman, A. L. (2006) Comparative genomics of NAD biosynthesis in cyanobacteria. *J. Bacteriol.* **188**, 3012–23

45. Rajendran, C., Gerhardt, E. C. M., Bjelic, S., Gasperina, A., and Scarduelli, M. (2011) Crystal structure of the GlnZ-DraG complex reveals a different form of P II -target interaction. *Proc. Natl. Acad. Sci.* 10.1073/pnas.1108038108/-/DCSupplemental.www.pnas.org/cgi/doi/10.1073/pnas.1108038108

46. Van Den Berg, S., Löfdahl, P. Å., Härd, T., and Berglund, H. (2006) Improved solubility of TEV protease by directed evolution. *J. Biotechnol.* **121**, 291–298

47. Araújo, L. M., Huergo, L. F., Invitti, A. L., Gimenes, C. I., Bonatto, A. C., Monteiro, R. A., Souza, E. M., Pedrosa, F. O., and Chubatsu, L. S. (2008) Different responses of the GlnB and GlnZ proteins upon in vitro uridylylation by the Azospirillum brasilense GlnD protein. *Brazilian J. Med. Biol. Res.* **41**, 289–294

48. Frickey, T., and Lupas, A. (2004) CLANS: A Java application for visualizing protein families based on pairwise similarity. *Bioinformatics*. 10.1093/bioinformatics/bth444

49. Rippka, R., Deruelles, J., and Waterbury, J. B. (1979) Generic assignments, strain histories and properties of pure cultures of cyanobacteria. *J. Gen. Microbiol.* **111**, 1–61

50. Pedrosa, F. de O., and Yates, M. G. (1984) Regulation of nitrogen fixation (nif) genes of Azospirillum brasilense by nifA and ntr (gln) type gene products. *FEMS Microbiol. Lett.* **23**, 95–101

51. Huergo, L. F., Chubatsu, L. S., Souza, E. M., Pedrosa, F. O., Steffens, M. B. R., and Merrick, M.

(2006) Interactions between PII proteins and the nitrogenase regulatory enzymes DraT and DraG in Azospirillum brasilense. *FEBS Lett.* **580**, 5232–6

52. Rocco, C. J., Dennison, K. L., Klenchin, V. A., Rayment, I., and Escalante-Semerena, J. C. (2008) Construction and use of new cloning vectors for the rapid isolation of recombinant proteins from Escherichia coli. *Plasmid*. **59**, 231–237

53. Moure, V. R., Razzera, G., Araújo, L. M., Oliveira, M. a S., Gerhardt, E. C. M., Müller-Santos, M., Almeida, F., Pedrosa, F. O., Valente, A. P., Souza, E. M., and Huergo, L. F. (2012) Heat stability of Proteobacterial PII protein facilitate purification using a single chromatography step. *Protein Expr. Purif.* **81**, 83–8

54. Araujo, M. S., Baura, V. A., Souza, E. M., Benelli, E. M., Rigo, L. U., Steffens, M. B. R., Pedrosa, F. O., and Chubatsu, L. S. (2004) In vitro uridylylation of the Azospirillum brasilense N-signal transducing GlnZ protein. *Protein Expr. Purif.* **33**, 19–24

55. Huergo, L. F., Souza, E. M., Araujo, M. S., Pedrosa, F. O., Chubatsu, L. S., Steffens, M. B. R., and Merrick, M. (2006) ADP-ribosylation of dinitrogenase reductase in Azospirillum brasilense is regulated by AmtB-dependent membrane sequestration of DraG. *Mol. Microbiol.* **59**, 326–37
